# Soil Fungal Communities Investigated by Metabarcoding Within Simulated Forensic Burial Contexts

**DOI:** 10.3389/fmicb.2020.01686

**Published:** 2020-07-24

**Authors:** Noemi Procopio, Stefano Ghignone, Samuele Voyron, Marco Chiapello, Anna Williams, Andrew Chamberlain, Antonietta Mello, Michael Buckley

**Affiliations:** ^1^Manchester Institute of Biotechnology, The University of Manchester, Manchester, United Kingdom; ^2^Istituto per la Protezione Sostenibile delle Piante, CNR, Turin, Italy; ^3^Dipartimento di Scienze della Vita e Biologia dei Sistemi, Università degli Studi di Torino, Turin, Italy; ^4^School of Applied Sciences, University of Huddersfield, Huddersfield, United Kingdom; ^5^School of Natural Sciences, The University of Manchester, Manchester, United Kingdom

**Keywords:** microbial ecology, next-generation sequencing, fungal communities, necrobiome, post-mortem interval, vertebrate decomposition, forensic science

## Abstract

Decomposition of animal bodies in the burial environment plays a key role in the biochemistry of the soil, altering the balance of the local microbial populations present before the introduction of the carcass. Despite the growing number of studies on decomposition and soil bacterial populations, less is known on its effects on fungal communities. Shifts in the fungal populations at different post-mortem intervals (PMIs) could provide insights for PMI estimation and clarify the role that specific fungal taxa have at specific decomposition stages. In this study, we buried pig carcasses over a period of 1- to 6-months, and we sampled the soil in contact with each carcass at different PMIs. We performed metabarcoding analysis of the mycobiome targeting both the internal transcribed spacer (ITS) 1 and 2, to elucidate which one was more suitable for this purpose. Our results showed a decrease in the fungal taxonomic richness associated with increasing PMIs, and the alteration of the soil fungal signature even after 6 months post-burial, showing the inability of soil communities to restore their original composition within this timeframe. The results highlighted taxonomic trends associated with specific PMIs, such as the increase of the Mortierellomycota after 4- and 6-months and of Ascomycota particularly after 2 months, and the decrease of Basidiomycota from the first to the last time point. We have found a limited number of taxa specifically associated with the carrion and not present in the control soil, showing that the major contributors to the recorded changes are originated from the soil and were not introduced by the carrion. As this is the first study conducted on burial graves, it sets the baseline for additional studies to investigate the role of fungal communities on prolonged decomposition periods and to identify fungal biomarkers to improve the accuracy of PMI prediction for forensic applications.

## Introduction

The introduction of a cadaver in a terrestrial environment has a strong effect on the ecosystem, providing a nutrient-rich source of food that is consumed by plants and scavengers, and generating an impact on the overall soil microbial dynamics (Barton et al., 2013). Changes in the microbial community can be used as an indicator of the time elapsed since death (also known as post-mortem interval, PMI) of the carrion, particularly in case of cadavers exposed on the soil surface (Hawksworth and Wiltshire, 2015; Bellini et al., 2016; Javan et al., 2016; Javan and Finley, 2018; Metcalf, 2019). Evaluating the PMI is a key factor in forensic investigations as it can provide important information on the timing and hence circumstances of death. However, numerous taphonomic factors (Christensen et al., 2014) play a significant role in the decomposition of organic remains, making the correct estimation of the PMI more challenging and limited in its accuracy, particularly in the case of heavily decomposed remains (Swift, 2006). For this reason, there is a requirement for the development of additional tools to improve the estimation of the PMI, which could complement existing ones (for an overview of the methods available for PMI estimation using microbiota see Metcalf, 2019). Recently, several independent studies have demonstrated the applicability of this type of study to the estimation of PMI by investigating the effects of different hosts (e.g., decomposing human, Parkinson et al., 2009; Cobaugh et al., 2015; Finley et al., 2016; Metcalf et al., 2016b; Szelecz et al., 2018; swine, Pechal et al., 2013; Guo et al., 2016; Procopio et al., 2018a; or rodents, Metcalf et al., 2013, 2016b; Guo et al., 2016), different burial conditions (e.g., exposed on the surface, Parkinson et al., 2009; Metcalf et al., 2013, 2016b; Pechal et al., 2013; Cobaugh et al., 2015; Finley et al., 2016; Guo et al., 2016; Szelecz et al., 2018 or shallow burials, Finley et al., 2016; Procopio et al., 2018a), different environments (e.g., indoor laboratory environments, Metcalf et al., 2013, 2016b or outdoor environments in different geographical locations, Parkinson et al., 2009; Pechal et al., 2013; Cobaugh et al., 2015; Finley et al., 2016; Guo et al., 2016; Metcalf et al., 2016b; Procopio et al., 2018a; Szelecz et al., 2018), and different substrates (e.g., in/on the carcass, Metcalf et al., 2013, 2016b; Pechal et al., 2013; Guo et al., 2016; Szelecz et al., 2018 or in the cadaver-associated soil, Parkinson et al., 2009; Metcalf et al., 2013, 2016b; Cobaugh et al., 2015; Finley et al., 2016; Procopio et al., 2018a; Szelecz et al., 2018). In general, it has been shown that, despite the obvious differences between different experimental designs, some microbial taxa are regularly associated with specific decomposition stages, and that specific taxa appear or increase in abundance at selected time points post-mortem in a similar manner across several independent studies (Procopio et al., 2018a; Metcalf, 2019). These results motivate additional research to improve the methods and to explore the possibility of applying this methodology in medico-legal investigations (Metcalf, 2019).

Metabarcoding analysis of soil microbial communities has been the subject of numerous studies not only in soil ecology and biochemistry, but also in forensic science, particularly due to the advent of next-generation sequencing (NGS) technologies that allow the characterization of the soil microbiome for numerous applications (Zhou and Bian, 2018). Metabarcoding refers to the characterization of operational taxonomic units (OTUs) by targeting specific “barcodes” in the microbial genome (Orgiazzi et al., 2013, 2015), either the bacterial one (selectively amplifying the hypervariable regions of the16S rRNA gene, e.g., V4) (Klindworth et al., 2013) or the fungal one [targeting the internal transcribed spacer (ITS) genomic regions] (Schoch et al., 2012). These approaches usually involve DNA extraction from soil, amplification via polymerase chain reaction (PCR) of a genomic region of interest (e.g., the “barcode” region), sequencing of the resulting amplicons, bioinformatic processing of the information and taxonomic evaluation of the analyzed samples (Taberlet et al., 2012).

Using microbes to estimate PMI is particularly promising (Metcalf, 2019) and may have particular utility when the body is heavily decomposed (e.g., dry or skeletonised remains) due to the current difficulty in the estimation of a precise PMI for skeletonised remains. In fact, in those cases PMI estimation often relies on the morphological examination of the remains, thus lacking objectivity and quantifiability, and numerous variables (e.g., temperature, humidity, exposure to sun, presence/absence, and type of soil) can strongly affect and ultimately impair the correctness of the estimations (Buekenhout et al., 2018). However, only in a few cases were long time frames investigated via microbial metabarcoding (Cobaugh et al., 2015; Finley et al., 2016; Metcalf et al., 2016b; Procopio et al., 2018a), leaving gaps in current knowledge. In particular, most previous studies have focused principally on the bacterial communities, despite some of them showing the potential of investigating both the bacterial and the fungal communities (Parkinson et al., 2009; Metcalf et al., 2016b) to maximize the success of addressing PMI from a microbial perspective. Parkinson et al. (2009) performed a combined analysis of both bacterial and fungal communities in cadaver-associated soil beneath two human cadavers using terminal restriction fragment length polymorphism (T-RFLP) on the ITS region. They noted the presence of specific succession patterns during several decomposition stages, although the intrinsic limits of the analysis performed did not allow them to identify specific fungal taxa associated with the observed temporal trend. Chimutsa et al. (2015) specifically investigated the fungal community changes in tanks filled with garden soil and a decomposing domestic pig (*Sus scrofa*) leg positioned at three different depths and at three time points after burial (3, 28, and 77 days). The fungal communities were characterized using PCR and denaturing gradient gel electrophoresis (PCR-DGGE) of the 18S rRNA gene. They did not find any statistically significant shift in fungal community diversity or numerical dominance in either the presence or the absence of the carcass, leading the authors to recommend different approaches such as the use of NGS to overcome the limitations of their study. Metcalf et al. (2016b) carried out a wider study including both mice (in laboratory settings) and decomposing human bodies exposed in outdoor settings at the Sam Houston State University (Southeast Texas Applied Forensic Science Facility). They evaluated at different PMIs the succession of bacteria, archaea, and fungi both on skin and within the cadaver-associated soil, highlighting the presence of several patterns characteristic for specific bacterial and fungal succession in the grave soil analyzed at increasing PMIs. Fu et al. (2015) performed metabarcoding analyses of the fungal communities associated with the decomposition of rat carcasses at three selected time points, and found significant correlations between the time elapsed since death and the fungal succession on the carcasses. More recently, Fu et al. (2019) also performed a similar experiment on juvenile pigs both exposed in outdoor conditions (in China, during the summer period, sampled from day 0 to day 14 post-mortem) and left to decompose indoors as a proxy to estimate PMI from the fungal shift. Also in this case, they found trends between specific decomposition stages and specific fungal taxa, suggesting potential for this type of analysis to estimate PMI.

Due to the important effect that environmental variables can have on the decomposition rate (Metcalf, 2019), it is crucial to perform additional outdoor studies in different geographical locations in order to test the possibility of building a regression model for PMI estimation from metabarcoding analyses that may be suitable for a wide range of environmental conditions, and to include temperature records in the model to take into account also the seasonality within the same environment (Metcalf, 2019). It is unclear whether forensic metabarcoding studies that were done in other, warmer and drier environments will provide models that are accurate in Britain with its cooler and less seasonal climate, and this phenomenon limits the applicability of the results found by other groups in this country. Britain has a markedly colder climate than the sites where similar studies have been conducted so far (e.g., Texas or China) and therefore it is crucial to investigate the microbial changes associated with decomposition in this specific environment to potentially obtain new insights on the estimation of PMI. Furthermore, this type of study has never been conducted on burial scenarios before, but only on exposed cadavers on soil; this weakness strongly affects the potential application of results obtained by other groups when working on buried remains. For this reason, we performed an experimental shallow outdoor burial of four pig carcasses at a rural site within Britain and investigated PMIs ranging from 1 to 6 months. In particular, we collected soil samples from the graves after 1, 2, 4, and 6 months post-mortem and conducted metabarcoding analyses on the samples. Our primary aim was to obtain a complete profile of the fungal populations associated with specific PMIs, to understand the impact that a buried carcass has on the soil biodiversity in a climate such as the British one and to evaluate if similar fungal successions were observed in different climates and for exposed cadavers. Secondary aims were to evaluate if there were differences in terms of the performance of the ITS1 and ITS2 regions of the fungal genome in this study, and ultimately to make recommendations for the design of similar experiments in other environmental conditions in the future.

## Materials and Methods

### Experimental Design

The experimental design of the study has been already described in detail in Procopio et al. (2018a), and is summarized here. Four juvenile pigs (*Sus scrofa*) (named P1, P2, P3, and P4) were buried under approximately 40 cm soil at the HuddersFIELD outdoor taphonomy facility (University of Huddersfield, United Kingdom) between May and November 2016. The experiment was conducted in accordance with the guidelines of the United Kingdom Department for Environment, Food and Rural Affairs (DEFRA). Animals used for this study died from natural causes prior to the experiment and were not euthanized for the purpose of the research. Soil physicochemical and biological characteristics on a control sample taken at approximately 40 cm in depth prior to the burials were undertaken by the C.N.R. – Institute for the Study of the Ecosystems, Pisa, Italy. Sample characteristics were electrical conductivity (0.53 dS m^–1^), total N (0.448%), total organic C (6.29%), total P (1.95 g/kg), available P (62.93 mg/kg), NO^3–^ (69.84 mgNO^3–^/kg), NH_3_ (1.93 mgNH3/kg), dehydrogenase activity (0.96 μgINTF/gss^∗^h), β-glucosidase activity (34 μmolMUB/g^∗^h), phosphatase activity (31 μmolMUB/g^∗^h), arylsulfatase activity (314 μmolMUB/g^∗^h), protease activity (15 μmolAMC/g^∗^h). Pigs of similar age (3–5 weeks old) and weight (4.5–11 kg), which had died from natural causes were kept frozen after death until their burial. Despite the freeze-thawing process having been shown to have an effect on the anaerobic bacterial communities of frozen/thawed mice allowed to decompose on the surface (Hyde et al., 2017), difference in body size between mice and pigs and in the depositional environment chosen (surface *versus* burial) make these findings potentially not applicable to our study. Furthermore, the effects of changes in the bacterial biota on the fungal community are not known, so despite there may be a change in the fungal community (due to the competition of bacteria and fungi for the same carcass), no one has demonstrated whether this actually occurs. The experiment time was set to zero when the cadavers were allowed to de-frost and were placed within soil graves, and subsequently each pig was left in the grave for a specific amount of time post-mortem (P1 = 1 month, P2 = 2 months, P3 = 4 months, and P4 = 6 months). The decomposition stages and the morphological aspects of the carcasses were evaluated and described in Procopio et al. (2018a). Briefly, after 1 month, P1 was in putrefaction, but overall the carcass shape was still recognizable; after 2 months, P2 was in the putrefaction/liquefaction stage of decomposition with bones still attached to ligaments; after 4 months, P3 was partially skeletonised with still some soft tissues present; after 6 months, P4 was fully skeletonised and disarticulated.

At each selected PMI, the graves were excavated and the carcasses sampled to collect soil samples for the metabarcoding. Soil samples in contact with the superior surface of the carcass (taken from the fore-limb area, the abdominal area and the hind-limb area of the body to maximize the microbial varieties sampled, e.g., taking into account potential microbial differences associated with different anatomical areas) were pooled in one bag per individual/burial at the selected PMI, and frozen at −20°C after their collection until further analysis. Three technical replicates per bag (i.e., one bag per grave) were subsampled from each bag and used for metabarcoding analyses. Additionally, control soil samples (taken at similar soil depths but without a carcass being present) were collected at locations 2 and 6 m away from the graves in order to exclude microbial contaminations from the graves. The control samples were obtained concurrently with the last sampling performed in November 2016 (6 months PMI), to provide a representative record of the background microbial activity within the field used for the experiment without direct microbial contamination from the graves. According to Metcalf et al. (2016b), the observable differences within the microbial community associated with the close proximity of a decomposing body are larger and more significant than those caused by variations in local environmental conditions (linked with seasonality, temperature, and rainfall). For this reason, our control samples were collected at different distances from the graves but at a single time point. Control soil samples were frozen at −20°C until further analysis. Metabarcoding samples totaled 18 (three samples per grave plus six controls). Soil pHs were recorded and reported in Procopio et al. (2018a), as well as soil temperatures and local temperatures and rainfalls (Procopio et al., 2018b).

### DNA Extraction, Amplification, and Sequencing

In order to characterize the fungal communities in the most efficient and reliable way, we targeted both ITS1 and ITS2 according to Mello et al. (2011). In fact, previous works showed contrasting results when dealing with ITS1 or ITS2. While Blaalid et al. (2013) obtained similar results when using either ITS1 or ITS2 to perform fungal metabarcoding with environmental data, Banchi et al. (2018b) showed discrepancies and biases in the detection of Basidiomycota when ITS1 or ITS2 were used as metabarcode for lichen mycobiomes, and suggested the complementary analysis of both ITS1 and ITS2 to reliably estimate the taxonomic diversity of the samples. In similar studies, Wang et al. (2015) concluded conversely that ITS1 provides a better DNA barcode than ITS2 allowing a better species discrimination efficiency, whereas the ITS2 has been specifically selected and preferred to ITS1 in another study to catch fungal diversity in airborne samples (Banchi et al., 2018a). It is clear that an *a priori* choice of the region/s to target was not ideal, and for this reason, our analysis of the fungal communities targeted specifically both the ITS1 and the ITS2 regions.

Genomic DNA was extracted from 500 mg of soil sample using the FastDNA SPIN Kit for Soil (MP Biomedicals, Europe), following the guidelines provided by the manufacturer, and fungal communities were specifically targeted amplifying the ITS regions in the nuclear ribosomal repeat unit ITS1 and ITS2. Forward ITS1-F (CTTGGTCATTTAGAGGAAGTAA) and reverse ITS2 (GCTGCGTTCTTCATCGATGC) primers for ITS1 (Gardes and Bruns, 1993) and forward fITS9 (GAACGCAGCRAAIIGYGA) and reverse ITS4 (TCCTCCGCTTATTGATATGC) primers (Sigma-Aldrich, United Kingdom) for ITS2 (Ihrmark et al., 2012 and White et al., 1990, respectively) were used in combination with 8 bp unique tags (Supplementary Table S1). The expected amplicon size was ∼250–600 bp for ITS1 (Bokulich and Mills, 2013; Hoggard et al., 2018) and ∼240–460 for ITS2. Primers and tags used for the subsequent analyses both for ITS1 and for ITS2 amplifications have been listed in Supplementary Table S2. Degeneracies were allowed in the primers to decrease eventual biases and to increase the ability to detect organisms characterized by slight modifications in their binding site’s sequences. PCR negative controls were run in each analysis to perform a quality check of the amplifications and in all cases these controls gave negative results. PCR reaction mixtures were set up as follows: 12.5 μL master mix (Platinum Hot Start PCR Master Mix 2×, Thermo Fisher, United Kingdom), 0.5 μL forward primer (10 μM), 0.5 μL reverse primer (10 μM), and 0.5–1.5 μL (2.0–5.0 ng/μL) template DNA in a final reaction volume of 25 μL. The thermal cycler (T3000, Biometra GmbH, Göttingen, Germany) conditions were set up as follows: denaturation at 94°C for 2 min; 35 cycles of denaturation at 94°C for 30 s, annealing at 52°C for 40 s, and extension at 68°C for 30 s; final extension at 68°C for 10 min and maintenance of the samples at 4°C. The PCR products obtained were checked on 1.5% (w/v) agarose gels (Sigma-Aldrich, United Kingdom), purified (Wizard SV Gel and PCR Clean-UpSystem, Promega, United Kingdom), quantified with Qubit (Qubit Fluorometric Quantitation, Thermo Fisher Scientific, United Kingdom) and sent to IGA Technology Services (Udine, Italy) for paired-end sequencing using the Illumina MiSeq technology (2 × 300 bp). The company ligated our tagged PCR products with a couple of Illumina adapters, and generated two libraries named “Library A” and “Library B” for ITS1 and ITS2, respectively, due to the different length of the amplicons expected for ITS1 and ITS2 as previously mentioned.

### DNA Data Analysis

Paired−end reads from each library were merged using Pear v.0.9.2 (Zhang et al., 2013), with the quality score threshold for trimming the low−quality part of a read set at 28, the minimum length of reads after the trimming process set at 200 bp and the minimum possible length of the assembled sequences set at 200 bp. Unix bash commands were used to select assembled sequences beginning with recognizable forward primer (no mismatch allowed), to trim initial and terminal 19 and 20 bases (corresponding to forward and reverse primers, respectively) and to assign a sample specific progressive count to each fragment. Assembled sequences from each library were clustered into OTUs using a *de novo* clustering strategy with QIIME v1.91 (Caporaso et al., 2010) and VSEARCH^1^ v2.3.4 (Rognes et al., 2016) at 97% similarity. Taxonomic assignment to OTUs was performed using the full “UNITE + INSD” dataset v.8.2 (released 2019-02-02; Nilsson et al., 2019) as a reference, and using BLAST and UCLUST, implemented in QIIME, as assignment methods. A consensus of the two assignment methods has been manually inspected and edited by mycologists and used for subsequent statistical analyses.

### Statistical Analyses

Numerical ecology statistical analyses were performed within the computing environment R^2^ (R Core Team, 2013). As previously described in Procopio et al. (2018a), control samples collected from two different locations were extracted in triplicate (replicate #1, #2, and #3) from each location (overall six DNA samples extracted), and during the data analysis they have been unified arbitrarily into a single control sample, summing up the reads of each of the three replicates to obtain our three replicates for control samples.

A number of filtering steps were applied to gather the final OTU dataset: OTUs with fewer than 50 reads were filtered out as well as samples with fewer than 20 reads and OTUs showing a coefficient of variation greater than 3.0 were also removed.

In order to standardize sampling efforts and to allow for comparisons of samples with non-uniform coverage, the OTU table has been normalized by subsampling at even sequencing depth from each sample using the minimal number of sequences obtained between the two libraries as value for the rarefaction (59,413 sequences) by means of the *rarefy_even_depth* function in the R package phyloseq V.1.22.3 (McMurdie and Holmes, 2013). The rarefaction effects on an OTU table are commonly rendered by means of the rarefaction curves, in order to graphically estimate species richness. Raw species richness counts can only be compared when the species richness has reached a clear asymptote; all the species present in a sample are well described when the curve ascribed to each sample reaches its plateau. Rarefaction curves were rendered by means of the function *ggrare*, provided by the richness. R script from the phyloseq extension package by Mahendra Mariadassou^3^. All taxon abundances were calculated and graphically plotted using the R package phyloseq v.1.28.0 (McMurdie and Holmes, 2013).

Biodiversity analyses were carried out by comparing the richness (number of species) and evenness (richness taking into account relative abundances) of microbial communities of the PMIs. Within-sample (alpha) diversity was assessed by six estimators: “observed number of species,” “Chao1,” “abundance-based coverage estimators (ACE),” “Shannon,” “Simpson,” “Fisher.” The alpha diversity indices were calculated and plotted by means of the functions *estimate_richness* and *plot_richness* implemented in the R package phyloseq (McMurdie and Holmes, 2013).

In order to test whether communities were statistically different from each other, a multivariate homogeneity of group dispersions among the different Control and PMI groups was first assessed by means of the *betadisper* and *permutest* (with 9,999 permutations) functions in the R package vegan V.2.5.2 (Oksanen et al., 2018). The differences in the composition of fungal communities in PMIs for both libraries were rendered by means of a nonmetric multidimensional scaling ordination (NMDS), to visualize Bray–Curtis distances, using the functions *vegdist* and *metaMDS* in the R package vegan (Oksanen et al., 2018).

The permutational multivariate analysis of variance (PERMANOVA, 9,999 permutations), as implemented in the *adonis* function of the vegan package of R (Oksanen et al., 2018), were applied to assess whether communities were statistically different from each other. Indicator species analysis [a classification-based method to measure associations between species and groups of sites (Dufrêne and Legendre, 1997)] was carried out using the *multipatt* function in the indicspecies v.1.7.6 R package, with 9,999 permutations (Cáceres and De Legendre, 2009) in order to assess whether OTUs (and if so, which ones) were significantly associated with a particular PMI.

To explore the shifts in the fungal basal communities at different decomposition stages, we evaluated which families showed statistically significant differences in the alpha diversity using the Kruskal–Wallis test and the Dunn’s pairwise tests, and in beta diversity and in the abundances at different PMIs applying the Student’s *t*-test with Benjamini and Hochberg False Discovery Rate (FDR) correction (Benjamini and Hochberg, 1995) with the T.TEST and P.ADJUST functions in R. This correction generated both *p* values and adjusted *p* values (*q* values), that were used to evaluate the statistical significance of the results reported in the work.

## Results

### Metabarcoding Analysis of Soil Fungi

Within the 18 samples analyzed, we respectively obtained 1,529,090 sequences after Library A pre-processing and 1,827,776 sequences after Library B pre-processing. Soil controls yielded overall 1,144,877 sequences (from 71,600 to 102,938 for Library A and from 46,536 to 141,094 for Library B), while grave soil samples yielded overall 2,211,989 sequences (from 60,085 to 113,214 for Library A and from 67,102 to 140,992 sequences for Library B). After the *de novo* clustering, sequences with 97% identity were clustered providing 1,640 initial OTUs for Library A and 2,569 initial OTUs for Library B. We identified overall two kingdoms within Library A and 12 within Library B (Supplementary Table S2), showing that the ITS2 pair of primers allowed wider amplification spectrum than the ITS1 pair. OTUs belonging to “Fungi” kingdom were respectively 99.7% in Library A (1,264 out of 1,268) and 81.8% in Library B (1,543 out of 1,885); all the OTUs belonging to different kingdoms were not considered for further analyses. Also, in terms of the number of reads, Library A showed slightly fewer reads for Fungi (∼1,500,000) than Library B (∼1,700,000), as well as fewer OTUs than Library B (Supplementary Figure S1). After the filtration step that removed OTUs whose cluster size was smaller than 50 reads (overall 601 OTUs for Library A and 857 OTUs for Library B were withheld after the filtration) and OTUs whose Coefficient of Variation was greater than 3.0, we obtained a final number of 571 OTUs for Library A and 749 OTUs for Library B (Supplementary Data S1). The sequence coverage obtained for both libraries showed adequacy for the proposed study (Supplementary Figure S2) and that, for both libraries, the control samples were the highest in terms of species richness, followed by P1, and then by P2–P4 that showed similarities to each other.

### Fungal Community Changes During Progressive Stages of Decomposition

**FIGURE 1:**
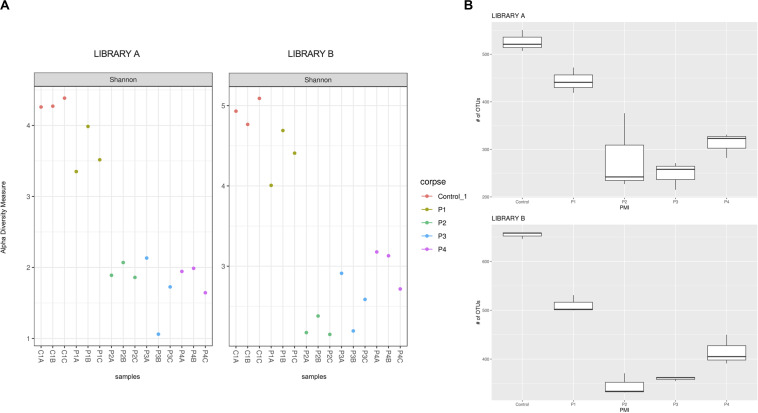
**(A)** Shannon alpha diversity measure of the samples collected after 1 (P1), 2 (P2), 4 (P3), and 6 (P4) months post-mortem for Library A (left) and Library B (right) and **(B)** box plot of the fungal richness of soil samples showing the raw number of OTUs per sample for Library A (top) and Library B (bottom), with standard deviations represented with vertical error bars and medians represented with a bold line in each box.

To account for richness and evenness of the microbial taxa associated with different PMIs, we calculated the Shannon–Wiener index and used boxplots to represent the species distribution among samples, with replicates grouped together in terms of raw species number (Figure 1). Other indices for alpha diversity such as Chao1, ACE, Simpson’s and Fisher’s index have been also applied, as recommended by Bandeira et al. (2013), in order to better define the communities (Supplementary Figure S3).

The Shannon–Wiener diversity indices (Figure 1A) showed statistical differences between the mean ranks of the groups for Library A and B (Kruskal–Wallis test, *p* = 0.027 and 0.012, respectively). However, after *post hoc* Wilcoxon test with Bonferroni correction the only statistical difference found was between controls and P2 for Library B (*q* = 0.014). We also calculated the linear regression models for both libraries (Supplementary Figure S4). The regression lines calculated for each library did not meet at any point in the graph, meaning that the Shannon–Wiener diversity indices for Library A and B were not statistically different from each other.

Taxonomic richness data were normally distributed (Shapiro–Wilk test, *p* = 0.13), and have been consequently evaluated using an analysis of variance (ANOVA) test (which showed a significant change for both libraries, *p* < 0.0001) followed by *post hoc* Tukey tests (Table 1).

**TABLE 1:**
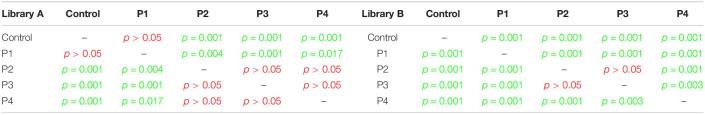
*Post hoc* Tukey test results for taxonomic richness calculated for Library A and B.

*Non-significant values have been reported in red. Significant values are reported in green.*

**FIGURE 2:**
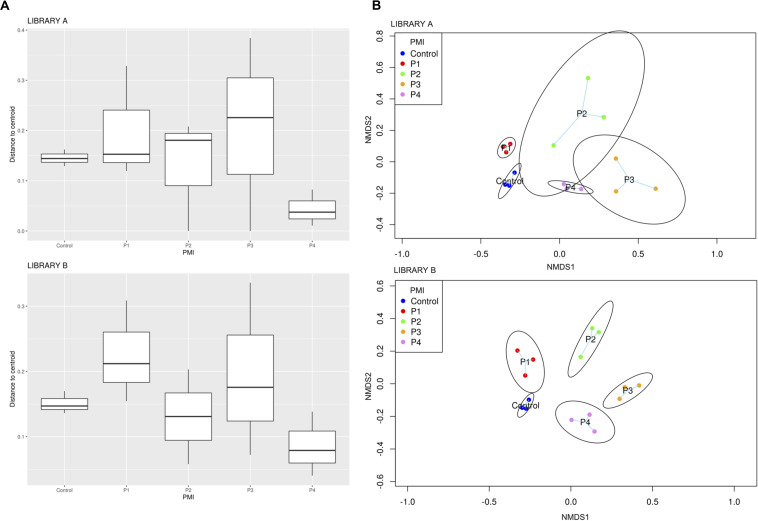
**(A)** Boxplot showing Bray-Curtis dissimilarity of the replicates from the centroid for specific PMIs for Library A (top) and Library B (bottom) and **(B)** NMDS plot showing an increase in beta diversity between the control soil and the grave soil, represented by expanded ellipses for P1, P2, P3, and P4 (P1 = 1 month, P2 = 2 months, P3 = 4 months, and P4 = 6 months). Standard deviation is represented by the dimension of the ellipses. Library A is at the top of the figure, and Library B at the bottom. The homogeneity of dispersion among groups was supported by a non-significant result in permutest (*p*-value = 0.48 for Library A and 0.31 for Library B).

Library B samples showed smaller standard deviations in the fungal richness (Figure 1B) and allowed for a better discrimination between the various time points than Library A. To test whether fungal communities were statistically different from each other, we evaluated the homogeneity of dispersion among groups (Figure 2A) and then ran a PERMANOVA test. The test provided significant results (*p* = 1e-04 for Library A and *p* = 1e-04 for Library B), thus indicating that the dissimilarity was influenced by difference in composition between groups. Consequently, we analyzed the differences in the taxonomic abundances from different samples to evaluate the beta diversity between different samples and we used a NMDS plot to visualize Bray-Curtis dissimilarities (Figure 2B). Samples collected from the same location clustered better in Library B, with the only exceptions being for P1 and P4, where the clusters were less scattered in Library A (Figure 2B). Overall, P1 was closer to the control, as previously reported also for bacterial data (Procopio et al., 2018a), whereas samples collected with increasing PMIs showed an ordered distribution from the control from the left to the right on the plot, with P2 being more distant, P3 being the most distant one and P4 being closer again to the control (Figure 2B).

To give a comparison of the NMDS ordinations of Bray–Curtis distances for Library A and B, we performed the Procrustes analysis that provides a plot where the same samples are connected by vector arrows. Results showed that the degree of concordance between Library A and B is statistically significant (*p* = 1e-04), meaning that the ordination of the data is similar between the two libraries (Supplementary Figure S5).

To characterize the differences found between the samples, we looked at the relative abundance of phyla (Supplementary Figure S6). Due to the incompleteness of the database for fungal species, we were not able to identify all of the species sequenced, and so we had to accept some unidentified phyla in the data.

**FIGURE 3:**
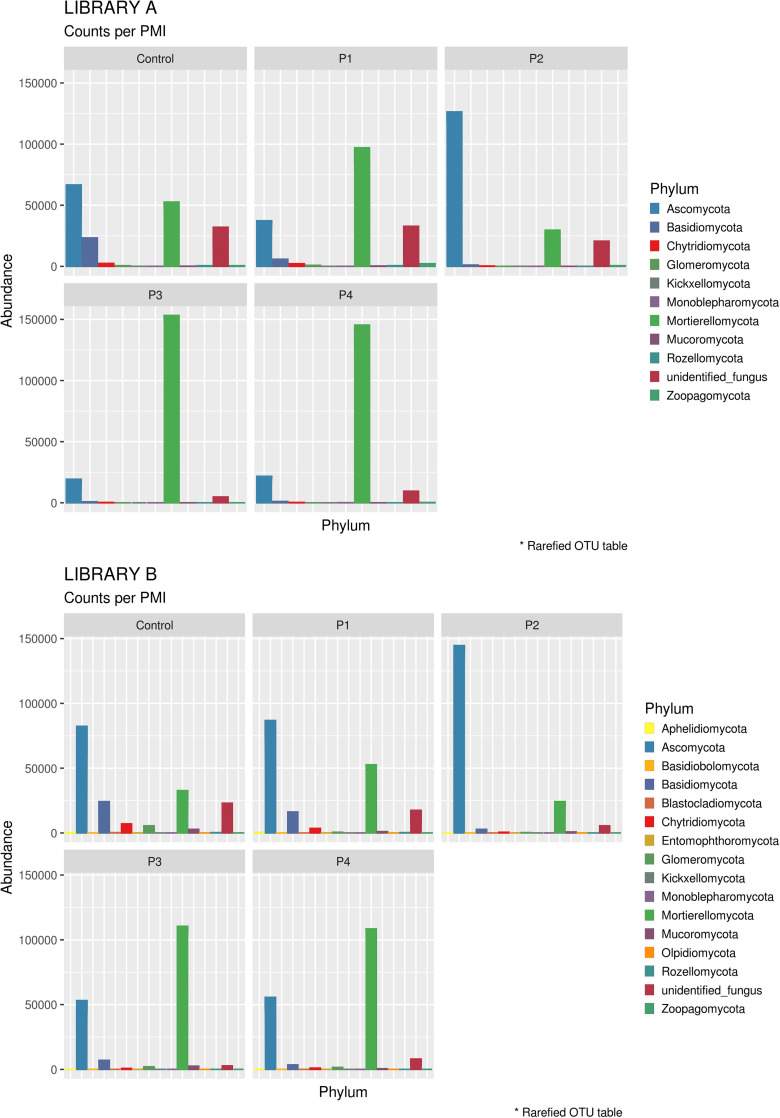
Bar chart with abundances of taxa at a phylum level associated with control soil and with the experimental samples in Library A **(top)** and Library B **(bottom)**. The order of the phyla represented in the legend is the same of the bars in the plot.

**FIGURE 4:**
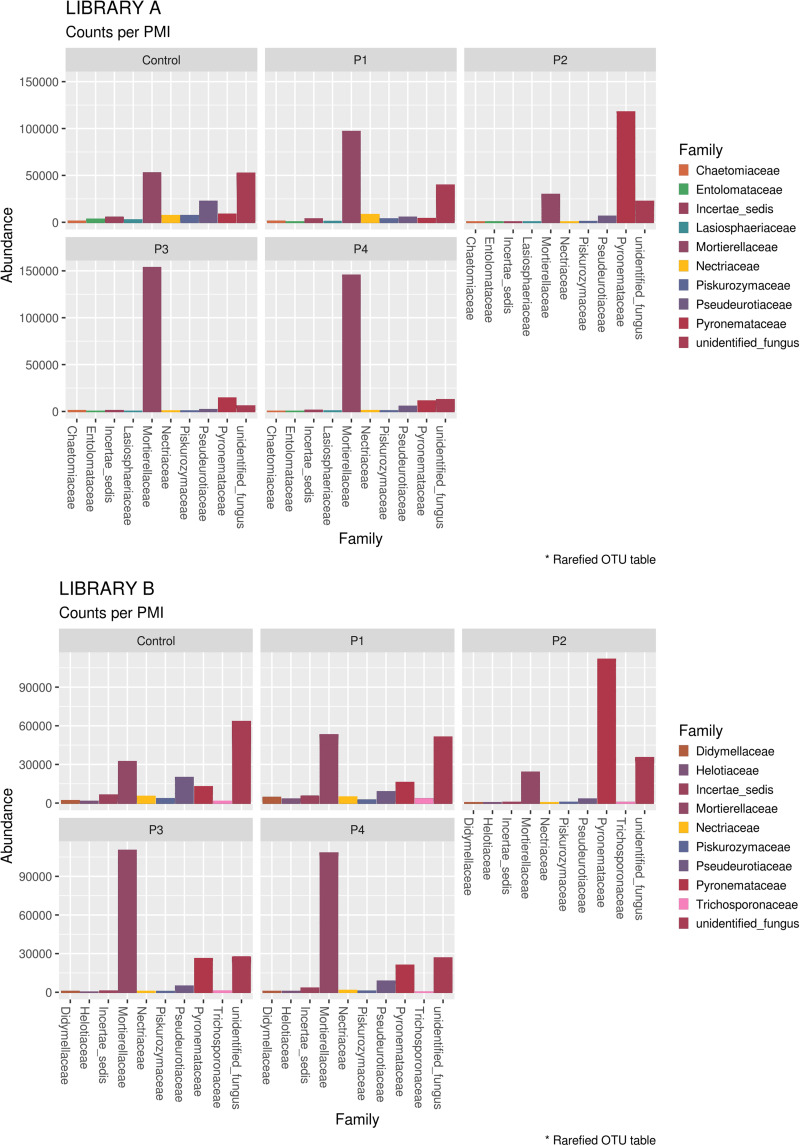
Bar chart with abundances of the top 10 taxa at a family level associated with control soil and with the experimental samples in Library A **(top)** and Library B **(bottom)**. The order of the families represented in the legend is the same of the bars in the plot.

When looking at specific PMIs, there were some noticeable trends with increasing time elapsed since death at phyla (Figure 3) and family level (Table 2, Figure 4, Supplementary Figure S7, and Supplementary Data S2). Ascomycota showed a notable increase in abundance after 2-months and a decrease after 4- and 6-months post-mortem compared with the controls for both libraries, despite their abundance decreasing after 2-months and slowly starting to increase again from 4-months onward. In particular, abundance of Pyronemataceae increased significantly in P2 in comparison with the control, and decreased after 4 months post-mortem. Abundance of Melanommataceae, Bionectriaceae, Massarinaceae, Onygenaceae, Pseudeurotiaceae, Coniochaetaceae, Gymnoascaceae, Chaetomiaceae, and Nectriaceae on the other hand showed a decline in P2, P3, and P4 compared with the control. Basidiomycota, with in particular Entolomataceae and Psathyrellaceae, constantly decreased with increasing PMIs. Mortierellomycota increased in abundance in P1 (in particular the Mortierellaceae family), then decreased in P2 and were the most abundant phylum at P3 and P4.

**TABLE 2:** Families grouped according to their phylum showing statistical differences between the control and the various time points (P1 to P4).

	Library A				Library B			
	P1	P2	P3	P4	P1	P2	P3	P4
**Ascomycota**								
*Massarinaceae*	↓ *p* = 0.004	↓ *p* = 0.0006	↓ *p* = 0.0007	↓ *p* = 0.001	↓ *p* = 0.002	↓ *p* = 0.0009	↓ *p* = 0.0008	↓ *p* = 0.005
*Pyronemataceae*	–	↑ *p* = 0.002	–	–	–	↑ *p* = 0.003	–	–
*Melanommataceae*	–	↓ *p* = 0.004	↓ *p* = 0.004	↓ *p* = 0.004	–	–	–	–
*Bionectriaceae*	–	↓ *p* = 0.002	↓ *p* = 0.003	↓ *p* = 0.002	–	–	–	–
*Onygenaceae*	–	↓ *p* = 0.002	–	–	–	–	–	–
*Pseudeurotiaceae*	–	–	–	–	–	↓ *p* = 0.004	↓ *p* = 0.005	–
*Coniochaetaceae*	–	–	–	–	–	↓ *p* = 0.003	↓ *p* = 0.003	↓ *p* = 0.005
*Gymnoascaceae*	–	–	–	–	–	↓ *p* = 0.004	–	–
*Chaetomiaceae*	–	–	–	–	–	↓ *p* = 0.003	–	↓ *p* = 0.004
**Zoopagomycota**								
*Piptocephalidaceae*	↑ *p* = 0.004	–	–	–	–	–	–	–
**Zygomycota**								
*Mortierellaceae*	–	–	–	↑ *p* = 0.003	–	–	–	–
**Basidiomycota**								
*Entolomataceae*	–	–	–	–	–	↓ *p* = 0.005	–	–
*Psathyrellaceae*	–	–	–	–	–	↓ *p* = 0.0009	↓ *p* = 0.0008	↓ *p* = 0.004
**Glomeromycota**								
*Diversisporaceae*	–	–	–	–	–	↓ *p* = 0.005	↓ *p* = 0.005	↓ *p* = 0.005

*Statistical significance was calculated via Student’s t-test with False Discovery Rate (FDR) correction. Results reported here were selected for p value ≤ 0.005. The symbol “↑” indicates an increase in comparison to the control, and the symbol “↓” a decrease. Non-significant values have been reported with the symbol “–.”*

### Identification of Endogenous Mammalian Fungal Communities and Basal Communities

**FIGURE 5:**
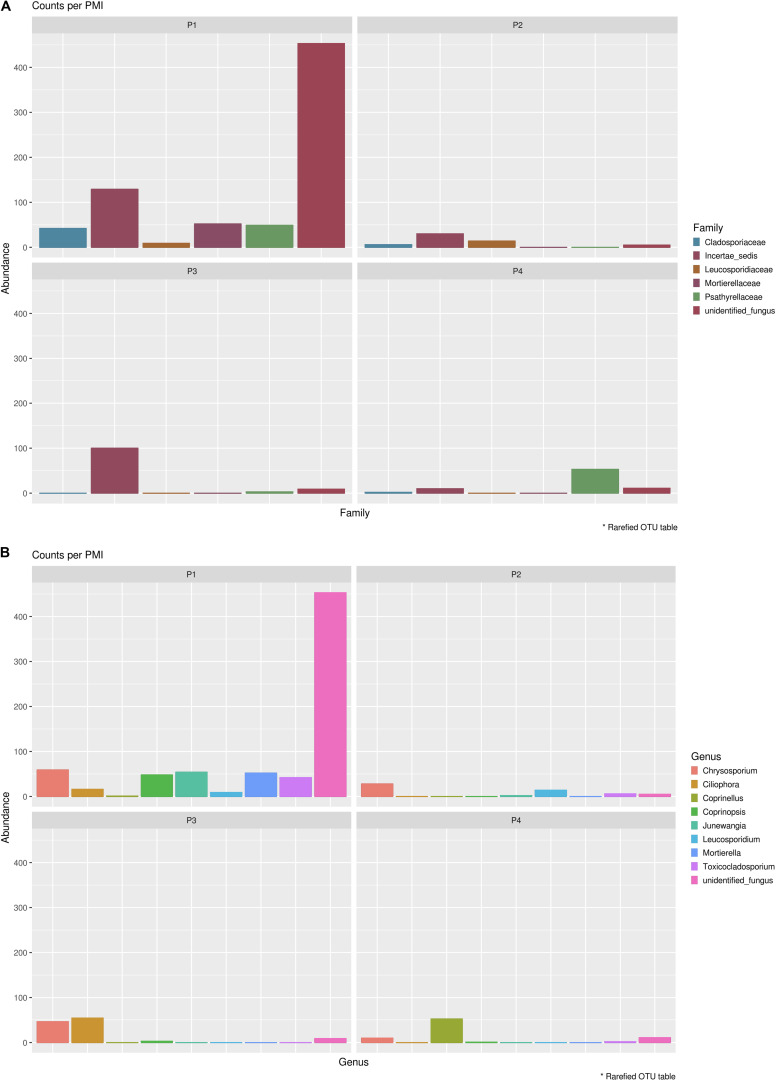
Mycobiome associated uniquely with the mammalian carcasses and not identified within the control sample at **(A)** family level and **(B)** genus level. Abundances per each family or genus were reported in different colors (see legend). The order of the families and genus represented in the legend is the same of the bars in the plot.

In order to identify specifically basal fungal communities that vary depending on the PMI of the bodies, excluding the taxa introduced in the cadaveric soil by the carcass (e.g., skin fungi present in and on the bodies), we evaluated which taxa were uniquely present in the cadaveric soil and not in the control samples, and then excluded these unique OTUs from the analysis of the “basal community.” In summary, for Library A we found 569 different OTUs present within the basal microbiome (Supplementary Data S3) and only two taxa specifically associated with the bodies (not identified in the control; Supplementary Data S4), and for Library B we found 735 different OTUs present within the basal microbiome (Supplementary Data S3) and 14 taxa specifically associated with the bodies (not identified in the control; Supplementary Data S4). It can be noted here that primers used for Library B allowed the identification of a higher number of fungi OTUs associated with the decomposing carcass than primers for Library A. For this reason, we will discuss results regarding the mycobiome introduced by the carcass only with reference to Library B (Figure 5). The results showed that the carcass’ fungal community that was abundant in soil at the first time point, almost disappeared with advanced decomposition stages, apart from some outliers such as the Onygenales order (family “Incertae sedis,” genus *Chrysosporium*) which were more abundant in P1 and P3, and the Psathyrellaceae (genus *Coprinellus*) which started to increase only after 6 months. The term “Incertae sedis” found in Figure 5 is a mycological term used to combine together different taxa which have not been classified yet into a specific family, and that for this reason can only be grouped in this way.

Indicator species analysis was also undertaken to evaluate which species were significantly associated specifically with the basal microbiome or with the carcasses (significance level = 5%) (Supplementary Data S5). Overall, the analysis showed that 253 OTUs for Library A and 295 species for Library B contributed to explain the differences observed between the control and the grave soil (*p* value ≤ 0.05), and that two OTUs contributed significantly to discriminate carrion-associated fungi from the basal fungal communities for Library B, but none for Library A. To report only taxa associated with significant increases or decreases at selected PMIs, we performed a Student’s *t*-test with FDR correction. Results obtained were almost identical to the ones showed in Table 2, with the same families having significant differences between the control and the various PMIs (Supplementary Figures S8–S11 and Supplementary Data S6).

## Discussion

The introduction of a decomposing carcass in the burial environment significantly alters the structure of the soil fungal communities, causing measurable changes such as an overall decrease in the richness of the fungal community with increasing PMIs. These findings are similar to what has been already found for bacteria in the burial environment (Procopio et al., 2018a), for fungal communities in soil underneath decomposing exposed pigs (Fu et al., 2019) and for bacterial communities present in different anatomical locations of human cadavers (Pechal et al., 2018). This result suggests that the presence of a decomposing carcass has a negative impact on the diversity of the fungal communities present in the soil, and the effect is particularly exacerbated when the body reaches the putrefaction/liquefaction stage of decomposition, where the least richness has been recorded. The biotic and abiotic factors in the soil environment are altered due to the release of organic compounds in the soil at different decomposition stages (Vass et al., 2004), and fungal species present in the environment as well as fungi originating from the microflora of the body are subjected to a growth inhibition and potentially to inter-specific competition that can result in sequential changes to the composition of the fungal community (Parkinson et al., 2009; Pechal et al., 2018). Fungal communities seem to be more sensitive than bacteria to alterations of soil environment, with significant reductions in species richness being already observed between the controls and the soil collected at the first time point, in contrast to what was observed for bacteria (Procopio et al., 2018a). Fungi also appear to start returning toward the original community structure (i.e., the one existing previously to the introduction of the carcass) faster than do the bacteria (Procopio et al., 2018a), as shown by the significant increase in the taxonomic richness of the samples collected after 6 months PMI. This finding could be due to their reduced sensitivity to strict pH parameters in comparison with bacteria (Finley et al., 2015) and to their potential better adaptation to new environments (Selbmann et al., 2013). Despite the quicker return to the original conditions for fungi compared to bacteria, more than 6 months appeared to be necessary for both fungi and bacteria to return to their original “basal” state; an observation that may be particularly useful in the investigation of clandestine movements of buried bodies from their original grave to a secondary grave.

We were able to identify specific trends for different phyla that might be good indicators for specific PMIs of buried cadavers. In particular, we found a significant increase in the abundance of Pyronemataceae (Ascomycota) after 2-months PMI that may be specific to buried cadavers. In fact, the increase of the abundance of Ascomycota during the first stages of decomposition was not reported by Fu et al. (2019) within soil collected underneath exposed pig cadavers. Ascomycota are decomposers able to break down the animal tissues into major organic compounds, and they have already been identified as fungal decomposers particularly active during the active decay of the carcass (Metcalf et al., 2016a). Recently, Bruns et al. (2020) have postulated that the post-fire microbial habitat is structured by a thermo-chemical gradient, which cause two main effects: a temperature gradient that causes differential mortality with soil depth, and a chemical gradient that structures resources available to recolonizing microbes. On this basis, we can postulate that the rise of temperature originated by the decomposition process in the burial environment that, even if lower than that caused by a fire, is extended in time (e.g., at least 2 months in our study), could cause the same effects, thus leading to the strong presence of fungi belonging to the family Pyronemataceae recorded here.

Basidiomycota on the other hand showed a constant decrease in their abundance starting from a greater amount and variety of families in control samples and ending in a significantly lower amount with prolonged PMIs. This result is in contrast with that of Metcalf et al. (2016a), who suggested that Basidiomycota are particularly abundant during the active decay stage, but is in agreement with Fu et al. (2015), who noticed a decrease in the abundance of this phylum in the rectum of decomposing rats with increasing time after death. In our case, the carcasses reached the active stage of decomposition between 1 and 2 months, but Basidiomycota decreased rapidly with the introduction of the carcass into the soil (from control to P1), with the Piskurozymaceae family being the most abundant one after 1 month, and with all the families, including Entolomataceae and Psathyrellaceae disappearing completely after 2, 4-, and 6-months post-mortem, suggesting their limited role during the active decomposition of the carcasses in this study. The same happened for the Glomeromycota phylum, where Diversisporaceae constantly decreased from P2 onward.

Mortierellomycota, and in particular the Mortierellaceae family, were previously found to be particularly active in between the active decay and the dry remains stages (Metcalf et al., 2016a). Our results are partially in agreement with this finding, with an increase of Mortierellaceae in P3 and P4, however, we also found an increase from the control to P1, suggesting that they also may have a role during the early decomposition stages of the carcasses, particularly when in the presence of buried carcasses.

We did not find an increase in Zygomycota and in Chytridiomycota with time, as found in contrast by Fu et al. (2015) in the rectum of decomposing rats, indicating that the soil fungal communities may act in a different way from the internal communities.

Unfortunately, the very limited amount of metabarcoding data available on fungal communities associated with cadaveric decomposition does not allow us to perform a comprehensive comparison between different studies. However, we found some similarities with the results of the study conducted by Metcalf et al. (2016a) on human cadavers in Texas, also with the one conducted by Fu et al. (2015) on rat carcasses in China and with the subsequent study conducted by the same group on exposed pigs (Fu et al., 2019), despite the fact that in all cases these studies were conducted with exposed remains instead of with buried ones as was the case in our study. So, it could be argued that the fungal succession during the various decomposition stages appears reproducible and reliable despite different geographical areas, different carrion species and different post-mortem conditions (e.g., buried vs non-buried). This highlights the potential for this type of analysis to provide a complementary fungal “clock of death” similar to the one already proposed for bacteria (Metcalf et al., 2013), provided that further studies are conducted in different geographical areas, with a higher number of timepoints and in different depositional environments in order to build a regression model that could assist in the prediction of the PMI.

Considering the fungal taxa introduced into the burial environment by the carcass, we noticed that the cadaveric soil is not a favorable environment for the survival and for the growth of these introduced species, and that overall fungi introduced by the body are replaced by soil fungi in less than 2 months. Similar findings were presented also by Fu et al. (2019), who showed that the variety of soil fungi present after 1-day post-mortem was higher than the one found during the decomposition of the carcasses. An example of this is represented by the Cladosporiaceae family. This family has been found to be a physiologic inhabitant of the intestine of the pig (Hurst, 2016), and our results showed that Cladosporiaceae species are not able to survive with advancing decomposition stages. Surprisingly, we did not find evidence in living pigs for the other families found to be abundant in P1, raising a question around the decrease, and then the eventual increase after several months, of some of these fungal families in the cadaveric soils. Interestingly, specific members of the Mortierellaceae family were found to be more abundant in P1 and then disappeared at prolonged PMIs. Although this may seem to contradict the previous findings, it should be noted that the specific taxa mentioned in this section of the work are the ones not found in the control soil and exclusively brought in by the carcass itself. So, despite some taxa of this family not being able to survive after prolonged PMIs, other members of the Mortierellaceae family already present in the soil showed the ability to take advantage of the organic nutrients released with the decomposition of the carcasses to become the most abundant species in the cadaveric soil after 4- and 6-months post mortem.

When looking for species significantly associated specifically with the basal microbiome or with the carcasses, only two OTUs were found to have contributed to discriminate carrion-associated fungi from the basal fungal communities, proving how limited the contribution to the soil fungal communities introduced by the carrion is, and supporting the potential to extend these findings to future applications involving the presence of other carrions (including humans).

In order to achieve fungal taxonomic identification, ITS1 and ITS2 loci are normally the standard sequences of choice, despite different authors having contrasting opinions regarding their efficiency and, in particular, about which one is the best performing one. When comparing the performance of ITS1 and ITS2 loci, we found sometimes specific results for the specific library investigated (e.g., some families were found to be statistically significant only for one of the two libraries), but the trends found were always in agreement between the two (e.g., Massarinaceae decreased constantly and significantly both for Library A and B). Overall results showed that ITS1 and ITS2 performed similarly in the characterization of the various samples, despite the lower variation between the replicates found for ITS2 in comparison with ITS1.

## Conclusion

This study has shown that shifts in the fungal communities present in the grave soil of pig carcasses, like the results for bacterial communities, have the potential to reveal information about the PMI of buried carcasses from 1- to 6-months post mortem. Here we were able to demonstrate that specific fungal families, such as the Mortierellaceae or the Massarinaceae, may be ideal for the estimation of the PMI, providing that additional research with additional bodies and time points is done to confirm the findings presented here. According to our results, the decrease of the abundance of Masssarinaceae families in cadaveric soil compared to a control soil may indicate the presence of a decomposing body, and the reduction in abundance of specific Ascomycota families such as Melanommataceae, Bionectriaceae, Pseudeurotiaceae, Coniochaetaceae, and Chaetomiaceae, Basidiomycota families such as Psathyrellaceae and Glomeromycota families such as Diversisporaceae may indicate the presence of a body in active or advanced decomposition stages. On the contrary, the presence of an increased abundance of Pyronemataceae in the soil, in comparison with a control, may indicate the presence of a body in the active decomposition stage, whereas an increase of Piptocephalidaceae might indicate the presence of a body at the beginning of its decomposition, and the increase of Mortierellaceae might indicate the presence of a body in an advanced decomposition stage. We have also shown here that the specific analysis of the ITS1 or the ITS2 sites provide similar results, but that the range of data obtained with ITS2 is generally wider when compared to ITS1, allowing for a better characterization of the distinct PMIs. For this reason, we recommend here that in future work either the combination of the two ITS sites, or exclusively the ITS2 region should be targeted instead of using the ITS1 only. Despite the constraints found, including the limited availability of metabarcoding fungal data in forensic casework and the lack of curated databases, we were able in this study to provide interesting insights on the fungal shifts in the burial environment in temperate climates, finding some good correlations also with the results of other experiments conducted on different carrion species and in different environments. Overall, this pilot study showed the great potential that fungal metabarcoding has for forensic investigations, and should be the starting point for additional research to improve the estimation of PMI through the analysis of changes in soil fungal communities.

## Author’s Note

This manuscript has been released as a pre-print at ResearchSquare (Procopio et al., 2020).

## Data Availability Statement

The datasets generated for this study can be found in the NCBI Sequence Read Archive (SRA-NCBI; https://www.ncbi.nlm.nih.gov/sra) under project accession number PRJNA594906 and BioSample accession numbers SAMN13816975–SAMN13816980.

## Ethics Statement

The animal study was reviewed and approved by the School of Applied Sciences, University of Huddersfield. The tissues (carcasses) used in the experiment were fallen stock (animals that had died of natural causes). National regulations (DEFRA) were adhered to in all aspects of the experiments. Pig burials took place on a facility with a DEFRA license for such activity. The work adhered to any other regulations that may apply to experimental work undertaken at the various universities where analyses were undertaken.

## Author Contributions

NP, AM, and MB designed the study. NP, AC, AW, and MB performed the experimental burial and the soil samplings. NP and AM performed the extraction and amplification of DNA. SG, SV, and MC performed the bioinformatic analysis of the results. NP, SG, and SV performed the data interpretation. NP was the major contributor in writing the manuscript. AC, MB, and AW performed the proofreading of the manuscript. All authors read and approved the final manuscript.

## Conflict of Interest

The authors declare that the research was conducted in the absence of any commercial or financial relationships that could be construed as a potential conflict of interest.

## Footnotes

1 https://github.com/torognes/vsearch

2 https://www.R-project.org/

3 https://github.com/mahendra-mariadassou/phyloseq-extended

## References

[B1] Banchi E. Ametrano C. G. Stanković D. Verardo P. Moretti O. Gabrielli F. (2018a). DNA metabarcoding uncovers fungal diversity of mixed airborne samples in Italy. *PLoS One* 13:e0194489. 10.1371/journal.pone.0194489 29558527 PMC5860773

[B2] Banchi E. Stankovic D. Fernández-Mendoza F. Gionechetti F. Pallavicini A. Muggia L. (2018b). ITS2 metabarcoding analysis complements lichen mycobiome diversity data. *Mycol. Prog.* 17 1049–1066. 10.1007/s11557-018-1415-4 30956650 PMC6428334

[B3] Bandeira B. Jamet J.-L. Jamet D. Ginoux J.-M. (2013). Mathematical convergences of biodiversity indices. *Ecol. Indic.* 29 522–528. 10.1016/j.ecolind.2013.01.028

[B4] Barton P. S. Cunningham S. A. Lindenmayer D. B. Manning A. D. (2013). The role of carrion in maintaining biodiversity and ecological processes in terrestrial ecosystems. *Oecologia* 171 761–772. 10.1007/s00442-012-2460-3 23007807

[B5] Bellini E. Ambrosio E. Zotti M. Nucci G. Gabbrielli M. Vanezis P. (2016). The usefulness of cadaveric fungi as an investigation tool. *Am. J. Forensic Med. Pathol.* 37:23. 10.1097/PAF.0000000000000210 26505227

[B6] Benjamini Y. Hochberg Y. (1995). Controlling the false discovery rate: a practical and powerful approach to multiple testing. *J. R. Stat. Soc. Ser. B* 51 289–300. 10.1111/j.2517-6161.1995.tb02031.x

[B7] Blaalid R. Kumar S. Nilsson R. H. Abarenkov K. Kirk P. M. Kauserud H. (2013). ITS1 *versus* ITS2 as DNA metabarcodes for fungi. *Mol. Ecol. Resour.* 13 218–224. 10.1111/1755-0998.12065 23350562

[B8] Bokulich N. A. Mills D. A. (2013). Improved selection of internal transcribed spacer-specific primers enables quantitative, ultra-high-throughput profiling of fungal communities. *Appl. Environ. Microbiol.* 79 2519–2526. 10.1128/AEM.03870-12 23377949 PMC3623200

[B9] Bruns T. D. Chung J. A. Carver A. A. Glassman S. I. (2020). A simple pyrocosm for studying soil microbial response to fire reveals a rapid, massive response by *Pyronema* species. *PLoS One* 15:e0222691. 10.1371/journal.pone.0222691 32130222 PMC7055920

[B10] Buekenhout I. Cravo L. Vieira D. N. Cunha E. Ferreira M. T. (2018). Applying standardized decomposition stages when estimating the PMI of buried remains: reality or fiction? *Aust. J. Forensic Sci.* 50 68–81. 10.1080/00450618.2016.1212268

[B11] Cáceres M. De Legendre P. (2009). Associations between species and groups of sites: indices and statistical inference. *Ecology* 90 3566–3574. 10.1890/08-1823.1 20120823

[B12] Caporaso J. G. Kuczynski J. Stombaugh J. Bittinger K. Bushman F. D. Costello E. K. (2010). QIIME allows analysis of high-throughput community sequencing data. *Nat. Methods* 7 335–336. 10.1038/nmeth.f.303 20383131 PMC3156573

[B13] Chimutsa M. Olakanye A. O. Thompson T. J. U. Ralebitso-Senior T. K. (2015). Soil fungal community shift evaluation as a potential cadaver decomposition indicator. *Forensic Sci. Int.* 257 155–159. 10.1016/j.forsciint.2015.08.005 26322496

[B14] Christensen M. A. Passalacqua N. Bartelink E. (2014). “Forensic taphonomy,” in *Forensic Anthropology Current Methods and Practice*, ed. Rattenbury A. E. (Cambridge, MA: Academic Press), 119–147.

[B15] Cobaugh K. L. Schaeffer S. M. DeBruyn J. M. (2015). Functional and structural succession of soil microbial communities below decomposing human cadavers. *PLoS One* 10:e0130201. 10.1371/journal.pone.0130201 26067226 PMC4466320

[B16] Dufrêne M. Legendre P. (1997). Species assemblages and indicator species: the need for a flexible asymmetrical approach. *Ecol. Monogr.* 67 345–366. 10.1890/0012-9615(1997)067[0345:saaist]2.0.co;2

[B17] Finley S. J. Benbow M. E. Javan G. T. (2015). Potential applications of soil microbial ecology and next-generation sequencing in criminal investigations. *Appl. Soil Ecol.* 88 69–78. 10.1016/j.apsoil.2015.01.001

[B18] Finley S. J. Pechal J. L. Benbow M. E. Robertson B. K. Javan G. T. (2016). Microbial signatures of cadaver gravesoil during decomposition. *Microb. Ecol.* 71 524–529. 10.1007/s00248-015-0725-1 26748499

[B19] Fu X. Guo J. Finkelbergs D. He J. Zha L. Guo Y. (2019). Fungal succession during mammalian cadaver decomposition and potential forensic implications. *Sci. Rep.* 9:12907. 10.1038/s41598-019-49361-0 31501472 PMC6733900

[B20] Fu X. L. Guo J. J. Zhu Z. Y. Ding Z. Y. Zha L. Cai J. F. (2015). The potential use of fungi community in postmortem interval estimation in China. *Forensic Sci. Int. Genet. Suppl. Ser.* 5 e476–e478. 10.1016/J.FSIGSS.2015.09.189

[B21] Gardes M. Bruns T. D. (1993). ITS primers with enhanced specificity for basidiomycetes - application to the identification of mycorrhizae and rusts. *Mol. Ecol.* 2 113–118. 10.1111/j.1365-294X.1993.tb00005.x 8180733

[B22] Guo J. Fu X. Liao H. Hu Z. Long L. Yan W. (2016). Potential use of bacterial community succession for estimating post-mortem interval as revealed by high-throughput sequencing. *Sci. Rep.* 6:24197. 10.1038/srep24197 27052375 PMC4823735

[B23] Hawksworth D. L. Wiltshire P. (2015). Forensic mycology: current perspectives. *Res. Rep. Forensic Med. Sci.* 5 75–83. 10.2147/RRFMS.S83169

[B24] Hoggard M. Vesty A. Wong G. Montgomery J. M. Fourie C. Douglas R. G. (2018). Characterizing the human mycobiota: a comparison of small subunit rRNA. ITS1, ITS2, and large subunit rRNA genomic targets. *Front. Microbiol.* 9:2208. 10.3389/fmicb.2018.02208 30283425 PMC6157398

[B25] Hurst C. J. (ed.) (2016). *Advances in Environmental Microbiology.* Berlin: Springer.

[B26] Hyde E. R. Metcalf J. L. Bucheli S. R. Lynne A. M. Knight R. (2017). “Microbial communities associated with decomposing corpses,” in *Forensic Microbiol*, ed. Carter John D. O. (Hoboken, NJ: Wiley Sons Ltd), 245–273. 10.1002/9781119062585.ch10

[B27] Ihrmark K. Bödeker I. T. M. Cruz-Martinez K. Friberg H. Kubartova A. Schenck J. (2012). New primers to amplify the fungal ITS2 region - evaluation by 454-sequencing of artificial and natural communities. *FEMS Microbiol. Ecol.* 82 666–677. 10.1111/j.1574-6941.2012.01437.x 22738186

[B28] Javan G. T. Finley S. J. (2018). “What is the ‘ Thanatomicrobiome’ and what is its relevance to forensic investigations?,” in *Forensic Ecogenomics*, ed. Ralebitso-Senior T. K. (Elsevier), 13–143.

[B29] Javan G. T. Finley S. J. Can I. Wilkinson J. E. Hanson J. D. Tarone A. M. (2016). Human thanatomicrobiome succession and time since death. *Sci. Rep.* 6:29598. 10.1038/srep29598 PMC4944132 27412051

[B30] Klindworth A. Pruesse E. Schweer T. Peplies J. Quast C. Horn M. (2013). Evaluation of general 16S ribosomal RNA gene PCR primers for classical and next-generation sequencing-based diversity studies. *Nucleic Acids Res.* 41:e1. 10.1093/nar/gks808 22933715 PMC3592464

[B31] McMurdie P. J. Holmes S. (2013). phyloseq: an R package for reproducible interactive analysis and graphics of microbiome census data. *PLoS One* 8:e61217. 10.1371/journal.pone.0061217 23630581 PMC3632530

[B32] Mello A. Napoli C. Murat C. Morin E. Marceddu G. Bonfante P. (2011). ITS-1 *versus* ITS-2 pyrosequencing: a comparison of fungal populations in truffle grounds. *Mycologia* 103 1184–1193. 10.3852/11-027 21700633

[B33] Metcalf J. L. (2019). Estimating the postmortem interval using microbes: knowledge gaps and a path to technology adoption. *Forensic Sci. Int. Genet.* 38 211–218. 10.1016/J.FSIGEN.2018.11.004 30448529

[B34] Metcalf J. L. Carter D. O. Knight R. (2016a). Microbiology of death. *Curr. Biol. Mag.* 26 R543–R576. 10.1016/j.cub.2016.03.042 27404249

[B35] Metcalf J. L. Xu Z. Z. Weiss S. Lax S. Van Treuren W. Hyde E. R. (2016b). Microbial community assembly and metabolic function during mammalian corpse decomposition. *Science* 351 158–162. 10.1126/science.aad2646 26657285

[B36] Metcalf J. L. Parfrey L. W. Gonzalez A. Lauber C. L. Knights D. Ackermann G. (2013). A microbial clock provides an accurate estimate of the postmortem interval in a mouse model system. *eLife* 2:e01104. 10.7554/eLife.01104 PMC3796315 24137541

[B37] Nilsson R. H. Larsson K.-H. Taylor A. F. S. Bengtsson-Palme J. Jeppesen T. S. Schigel D. (2019). The UNITE database for molecular identification of fungi: handling dark taxa and parallel taxonomic classifications. *Nucleic Acids Res.* 47 D259–D264. 10.1093/nar/gky1022 30371820 PMC6324048

[B38] Oksanen J. Blanchet F. G. Friendly M. Kindt R. Legendre P. McGlinn D. (2018). *Vegan: Community Ecology Package. R package version 2.4–6.*

[B39] Orgiazzi A. Bianciotto V. Bonfante P. Daghino S. Ghignone S. Lazzari A. (2013). 454 pyrosequencing analysis of fungal assemblages from geographically distant, disparate soils reveals spatial patterning and a core mycobiome. *Diversity* 5 73–98. 10.3390/d5010073

[B40] Orgiazzi A. Dunbar M. B. Panagos P. de Groot G. A. Lemanceau P. (2015). Soil biodiversity and DNA barcodes: opportunities and challenges. *Soil Biol. Biochem.* 80 244–250. 10.1016/J.SOILBIO.2014.10.014

[B41] Parkinson R. A. Dias K.-R. Horswell J. Greenwood P. Banning N. Tibbett M. (2009). “Microbial community analysis of human decomposition on soil,” in *Criminal and Environmental Soil Forensics*, eds Ritz K. Dawson L. Miller D. (Berlin: Springer), 379–394. 10.1007/978-1-4020-9204-6_24

[B42] Pechal J. L. Crippen T. L. Tarone A. M. Lewis A. J. Tomberlin J. K. Benbow M. E. (2013). Microbial community functional change during vertebrate carrion decomposition. *PLoS One* 8:e79035. 10.1371/journal.pone.0079035 24265741 PMC3827085

[B43] Pechal J. L. Schmidt C. J. Jordan H. R. Benbow M. E. (2018). A large-scale survey of the postmortem human microbiome, and its potential to provide insight into the living health condition. *Sci. Rep.* 8 1–15. 29636512 10.1038/s41598-018-23989-w PMC5893548

[B44] Procopio N. Ghignone S. Voyron S. Chiapello M. Williams A. Chamberlain A. (2020). *Soil Fungal Communities Investigated by Metabarcoding within Simulated Forensic Burial Contexts.* Preprint. Available online at: https://www.researchsquare.com/article/rs-13445/v1 10.3389/fmicb.2020.01686 PMC7393272 32793158

[B45] Procopio N. Ghignone S. Williams A. Chamberlain A. Mello A. Buckley M. (2018a). Metabarcoding to investigate changes in soil microbial communities within forensic burial contexts. *Forensic Sci. Int. Genet.* 39 73–85. 10.1016/j.fsigen.2018.12.002 30594064

[B46] Procopio N. Williams A. Chamberlain A. T. Buckley M. (2018b). Forensic proteomics for the evaluation of the post-mortem decay in bones. *J. Proteomics* 177 21–30. 10.1016/j.jprot.2018.01.016 29407476

[B47] R Core Team (2013). *R: A Language and Environment for Statistical Computing.* Vienna: R Core Team.

[B48] Rognes T. Flouri T. Nichols B. Quince C. Mahé F. (2016). VSEARCH: a versatile open source tool for metagenomics. *PeerJ* 4:e2584. 10.7717/peerj.2584 27781170 PMC5075697

[B49] Schoch C. L. Seifert K. A. Huhndorf S. Robert V. Spouge J. L. Levesque C. A. (2012). Nuclear ribosomal internal transcribed spacer (ITS) region as a universal DNA barcode marker for Fungi. *Proc. Natl. Acad. Sci. U.S.A.* 109 6241–6246. 10.1073/pnas.1117018109 22454494 PMC3341068

[B50] Selbmann L. Egidi E. Isola D. Onofri S. Zucconi L. de Hoog G. S. (2013). Biodiversity, evolution and adaptation of fungi in extreme environments. *Plant Biol.* 147 237–246. 10.1080/11263504.2012.753134

[B51] Swift B. (2006). “The timing of death,” in *Essentials of Autopsy Practice*, ed. Rutty G. N. (London: Springer), 189–214. 10.1007/b136465

[B52] Szelecz I. Lösch S. Seppey C. V. W. Lara E. Singer D. Sorge F. (2018). Comparative analysis of bones, mites, soil chemistry, nematodes and soil micro-eukaryotes from a suspected homicide to estimate the post-mortem interval. *Sci. Rep.* 8:25. 10.1038/s41598-017-18179-z 29311698 PMC5758714

[B53] Taberlet P. Prud’Homme S. Campione E. Roy J. Miquel C. Shehzad W. (2012). Soil sampling and isolation of extracellular DNA from large amount of starting material suitable for metabarcoding studies. *Mol. Ecol.* 21 1816–1820. 10.1111/j.1365-294X.2011.05317.x 22300434

[B54] Vass A. A. Smith R. R. Thompson C. V. Burnett M. N. Wolf D. A. Synstelien J. A. (2004). Decompositional odor analysis database. *J. Forensic Sci.* 49 760–769. 15317191

[B55] Wang X.-C. Liu C. Huang L. Bengtsson-Palme J. Chen H. Zhang J.-H. (2015). ITS1: a DNA barcode better than ITS2 in eukaryotes? *Mol. Ecol. Resour.* 15 573–586. 10.1111/1755-0998.12325 25187125

[B56] White T. J. Bruns T. Lee S. Taylor J. (1990). “Amplification and direct sequencing of fungal ribosomal RNA genes for phylogenetics,”in *PCR Protocols: A Guide to Methods and Applications*, eds Innis M. A. Gelfand D. H. Sninsky J. J. White T. J. (Academic Press), 315–322. 10.1016/B978-0-12-372180-8.50042-1

[B57] Zhang J. Kobert K. Flouri T. Stamatakis A. (2013). PEAR: a fast and accurate illumina paired-end reAd mergeR. *Bioinformatics* 30 614–620. 10.1093/bioinformatics/btt593 24142950 PMC3933873

[B58] Zhou W. Bian Y. (2018). Thanatomicrobiome composition profiling as a tool for forensic investigation. *Forensic Sci. Res.* 3 105–110. 10.1080/20961790.2018.1466430 30483658 PMC6197100

