# Supplementary material for: Soil Fungal Communities Investigated by Metabarcoding Within Simulated Forensic Burial Contexts
Source: Front Microbiol. 2020 Jul 24;11:1686. doi: 10.3389/fmicb.2020.01686 (PMC7393272; doi:10.3389/fmicb.2020.01686)
Supplement: Supplementary file 7 [file Data_Sheet_7.docx]

Supplementary Material

# Supplementary Data

**Supplementary Data S1.** Supplementary OTU table with counts of the reads and taxonomic allocations after the filtering and the rarefaction processes for Library A (first sheet) and B (second sheet).

**Supplementary Data S2.** Statistical analyses showing shifts in the overall communities at different PMIs comparing control soil (C1) versus grave soils (P1, P2, P3 and P4) for Library A and B.

**Supplementary** **Data S3.** OTU table with counts of the reads and taxonomic allocation of OTUs uniquely present in the basal mycobiome for Library A and B.

**Supplementary Data S4.** OTU table with counts of the reads and taxonomic allocations of OTUs characteristic of the grave soil for Library A and B.

**Supplementary Data S5.** Multilevel pattern analysis showing species that significantly characterised the basal microbiome and the grave soils.

**Supplementary Data S6**. Statistical analyses showing shifts in the basal communities at different PMIs comparing control soil (C1) versus grave soils (P1, P2, P3 and P4) for Library A and B.

# Supplementary Tables and Figures

## Supplementary Tables

**Supplementary Table S1**. Sequences of tags and list of the tags used for the amplification of ITS1 and ITS2 for each sample.

| **Tag sequences** | | | |
| --- | --- | --- | --- |
| n.1 | ACACACAC | n.13 | AGACTATG |
| n.2 | ACAGCACA | n.14 | GCGTCAGC |
| n.3 | GTGTACAT | n.15 | TGACATCA |
| n.4 | TATGTCAG | n.16 | ACATGTGT |
| n.5 | TAGTCGCA | n.17 | GTACGACT |
| n.6 | TACTATAC | n.18 | ATGATCGC |
| n.7 | ACTAGATC | n.19 | ACGACGAG |
| n.8 | GATCGCGA | n.20 | CATCAGTC |
| n.9 | CGCTCTCG | n.21 | ATCAGTCA |
| n.10 | GTCGTAGA | n.22 | TCTACTGA |
| n.11 | GTCACGTC | n.23 | GATGATCT |
| n.12 | GACTGATG | n.24 | CTGCGTAC |
| **Samples and selected tags for ITS1 amplification (Library A)** | | | |
| P1A | Tag n.22 | P4A | Tag n.12 |
| P1B | Tag n.23 | P4B | Tag n.11 |
| P1C | Tag n.1 | P4C | Tag n.10 |
| P2A | Tag n.2 | C1A | Tag n.9 |
| P2B | Tag n.18 | C1B | Tag n.8 |
| P2C | Tag n.3 | C1C | Tag n.7 |
| P3A | Tag n.16 | C3A | Tag n.6 |
| P3B | Tag n.15 | C3B | Tag n.5 |
| P3C | Tag n.14 | C3C | Tag n.13 |
| **Samples and selected tags for ITS2 amplification (Library B)** | | | |
| P1A | Tag n.1 | P4A | Tag n.10 |
| P1B | Tag n.2 | P4B | Tag n.11 |
| P1C | Tag n.13 | P4C | Tag n.12 |
| P2A | Tag n.4 | C1A | Tag n.21 |
| P2B | Tag n.5 | C1B | Tag n.23 |
| P2C | Tag n.6 | C1C | Tag n.15 |
| P3A | Tag n.7 | C3A | Tag n.16 |
| P3B | Tag n.20 | C3B | Tag n.17 |
| P3C | Tag n.9 | C3C | Tag n.22 |

**Supplementary Table S2**. Kingdoms identified for Library A and B.

| **Library A** | **Library B** |
| --- | --- |
| **Kingdoms identified:** | |
| Chromista | Alveolata |
| Fungi | Bacteria |
| Unassigned | Cercozoa |
|  | Chromista |
|  | Fungi |
|  | Metazoa |
|  | Protista |
|  | Protozoa |
|  | Rhizaria |
|  | Rhodophyla |
|  | Viridiplantae |
|  | Unassigned |

## Supplementary Figures

**
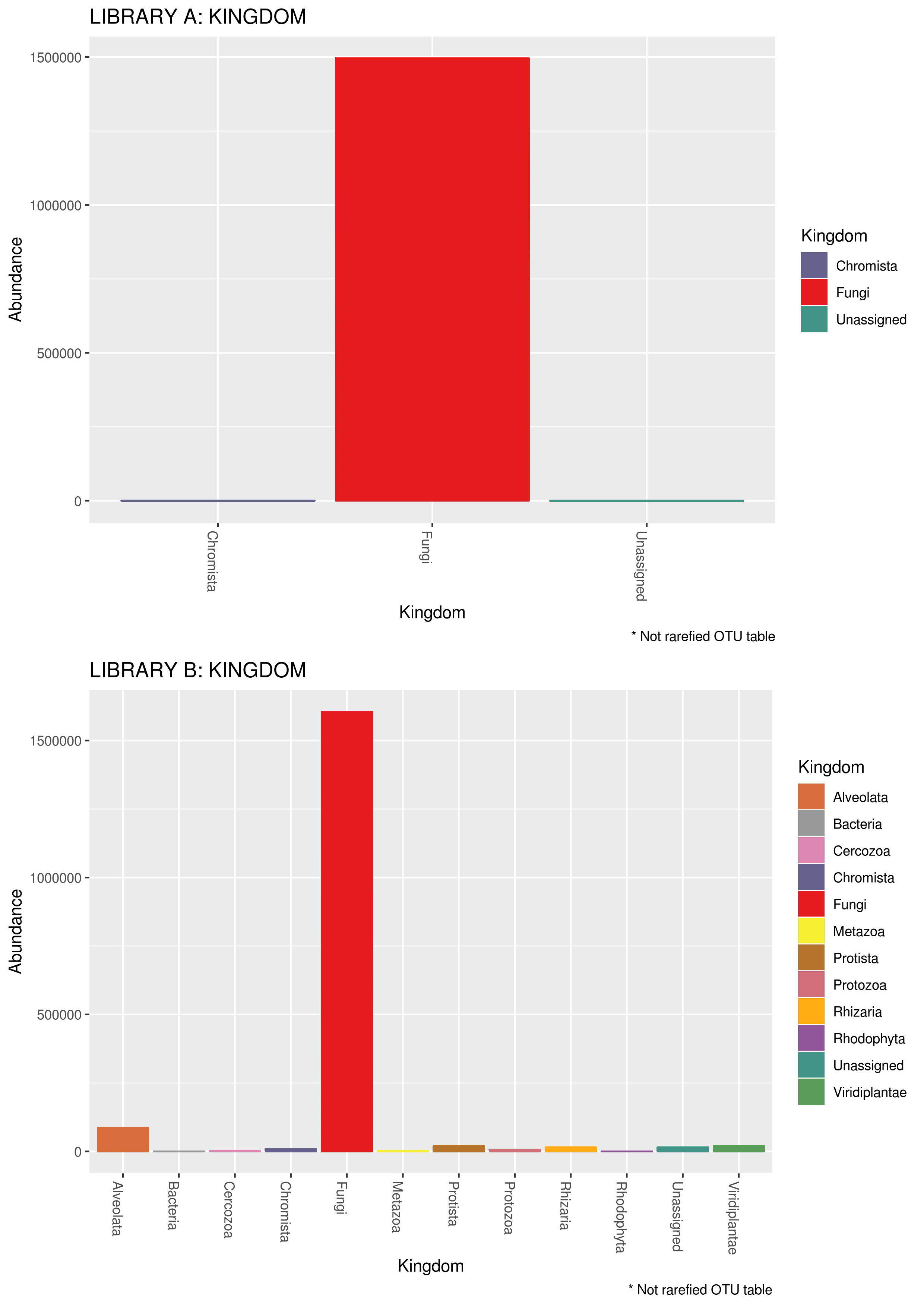
**

**Supplementary Figure S1.** Number of rarefied reads grouped per kingdoms identified in Library A (top) and B (bottom) before the filtration step.

**Supplementary Figure S2.** Rarefaction curves with estimation of the species richness per sample for Library A (top) and B (bottom).


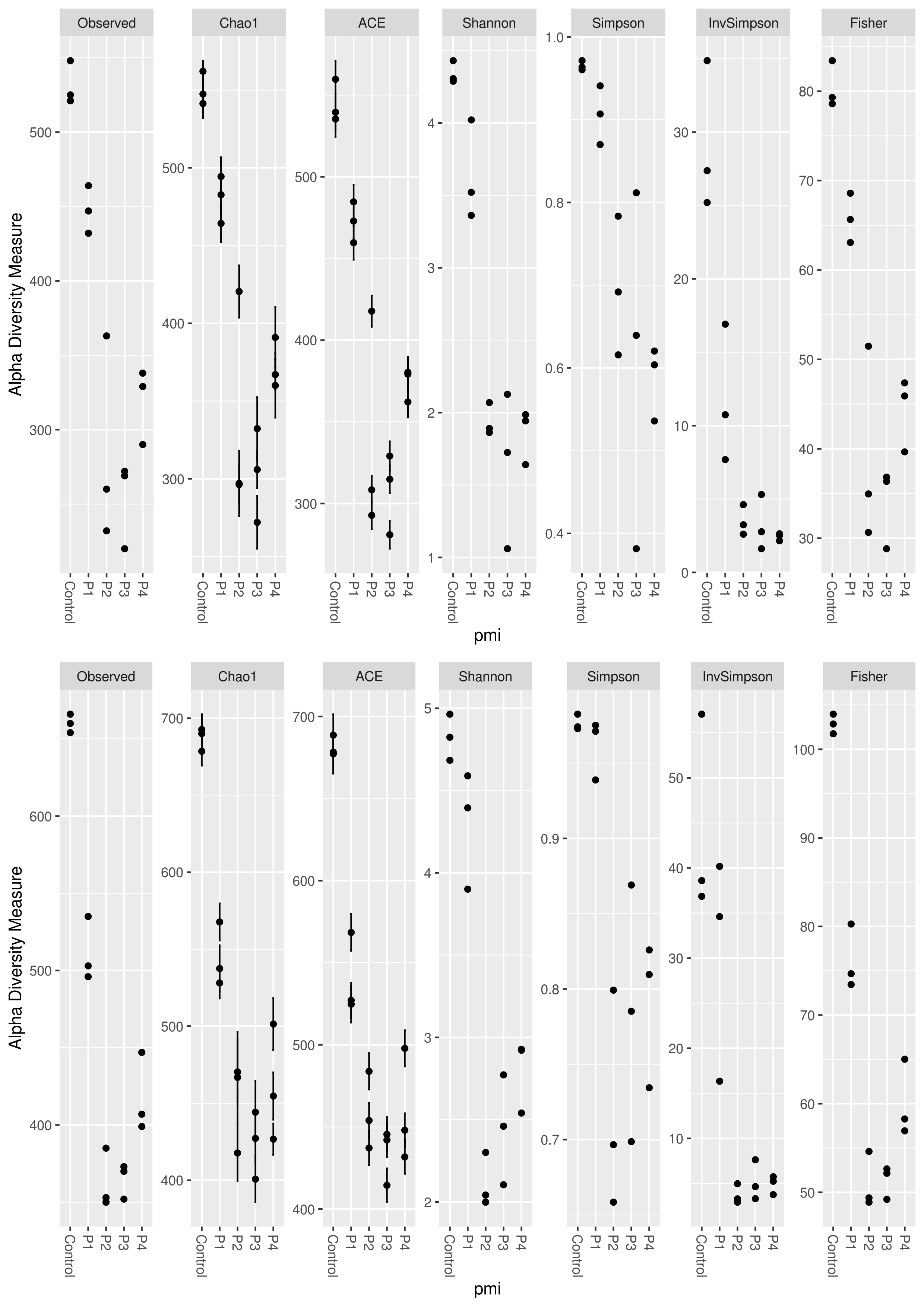


**Supplementary Figure S3.** Alpha diversity indices per sample evaluated in the study and grouped by PMI, including the observed species, Chao 1 index, ACE (abundance-based coverage estimators) index, Shannon index, Simpson and inverse Simpson indices and Fisher index for Library A (top) and B (bottom).


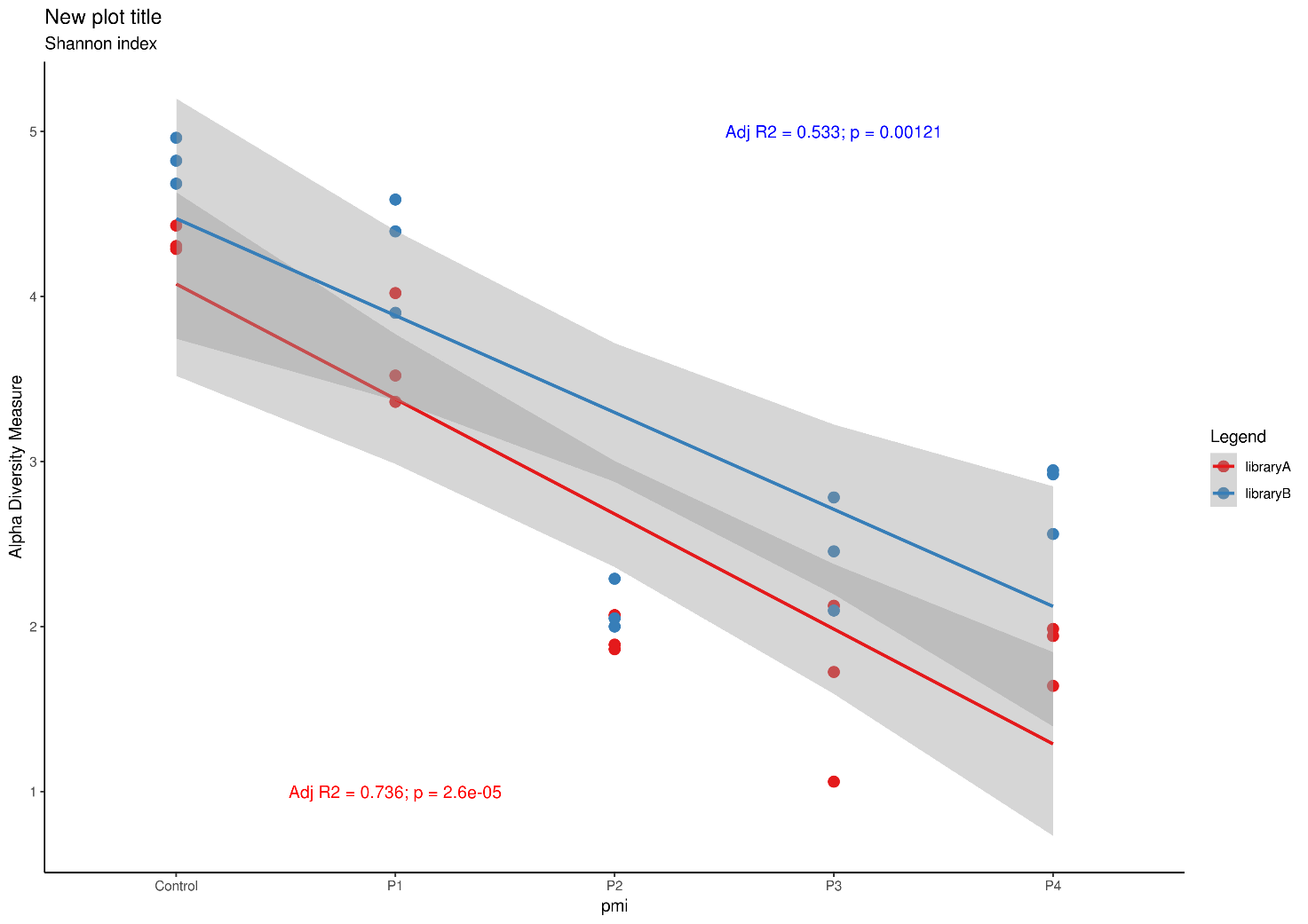


**Supplementary Figure S4**. Regression lines for Shannon-Wiener alpha diversity measures for Library A (red) and Library B (blue), and adjusted R2 and *p* values for both regression lines.

**Supplementary Figure S5**. Procrustes analysis showing the ordination of the samples according to their NMDS plots both for Library A (blue) and B (green). The same samples are connected by vector arrows (non-metric multidimensional scaling ordinations of Bray-Curtis distances). Vector length reflects the change in ordination space between the two libraries, and each of them points from the position of the samples in Library A towards their position in the Library B.


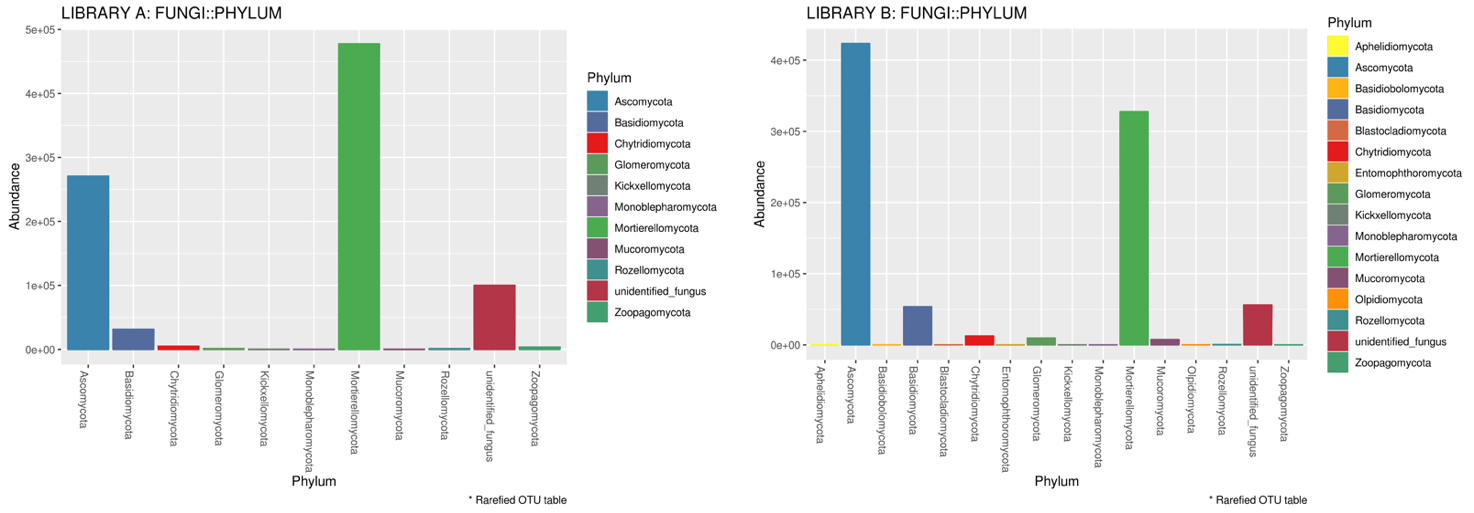


**Supplementary Figure S6**. Phyla abundances recorded overall in the experiment for Library A (left) and B (right).


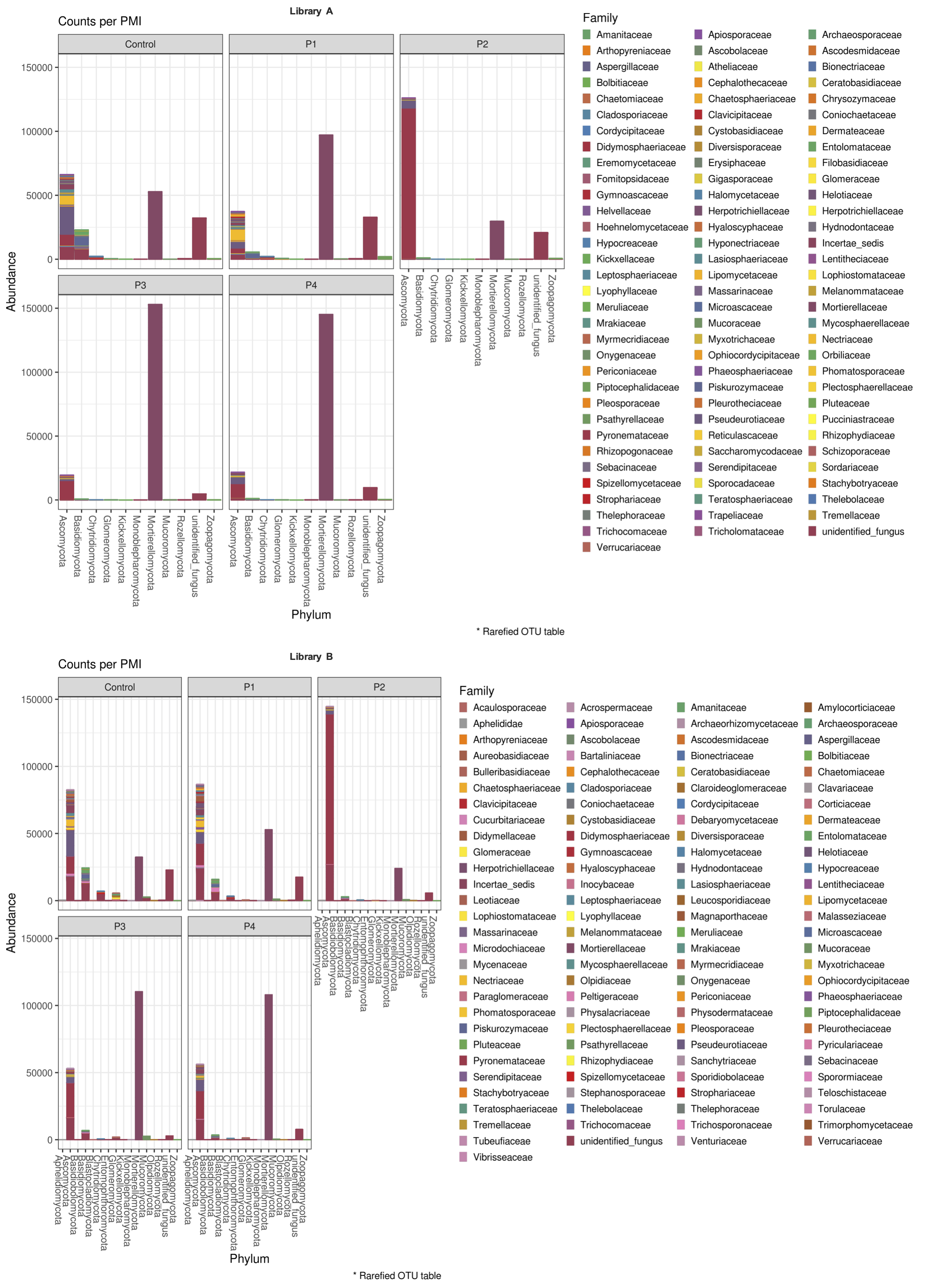


**Supplementary Figure S7.** Bar chart with abundances of taxa at a family level associated with control soil and with the experimental samples in Library A (top) and Library B (bottom).


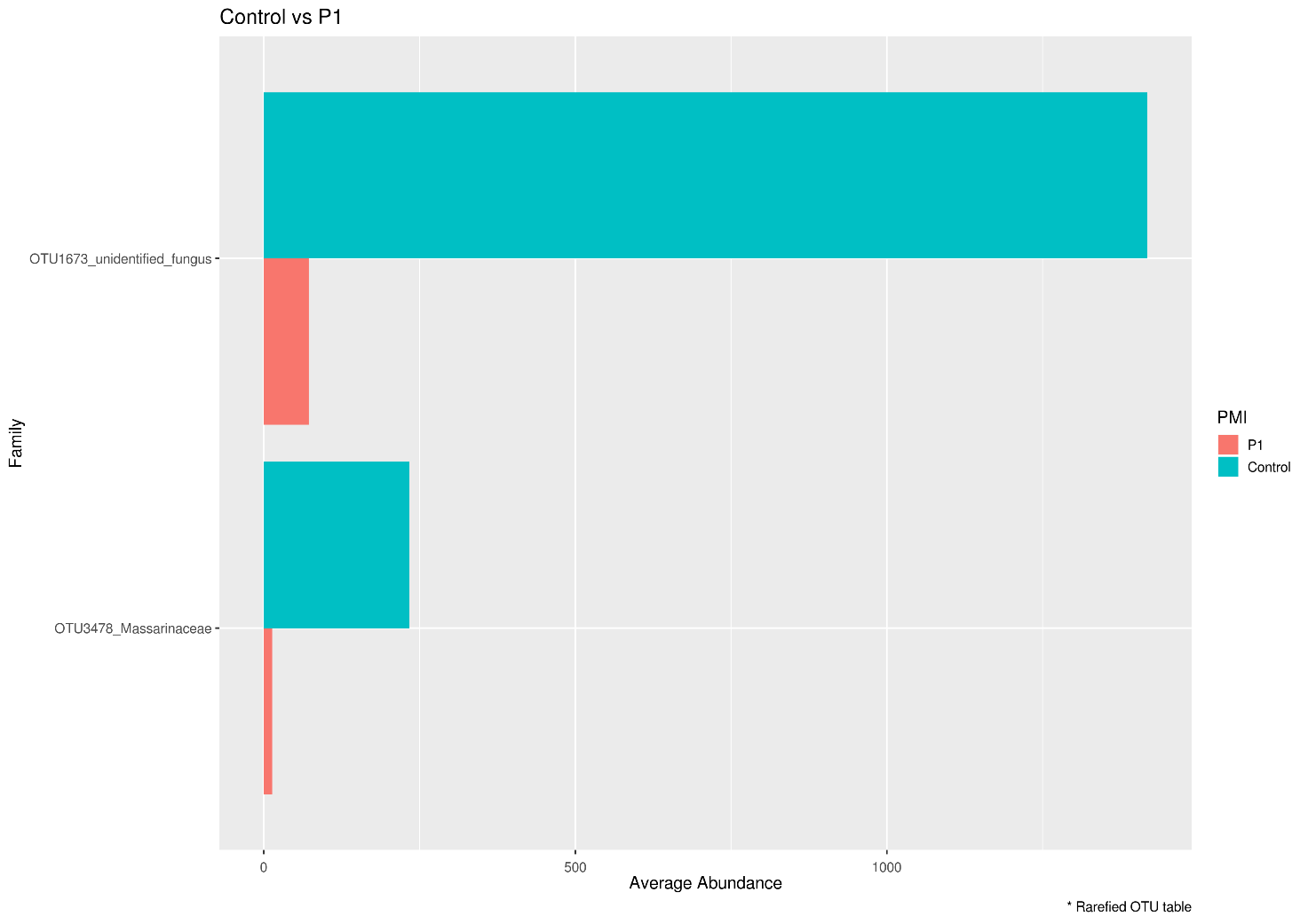


**Supplementary Figure S8**. Shifts in the average abundances of the basal communities that were statistically significant between the control (C1) and the grave soil collected after one-month PMI (P1) in Library B.


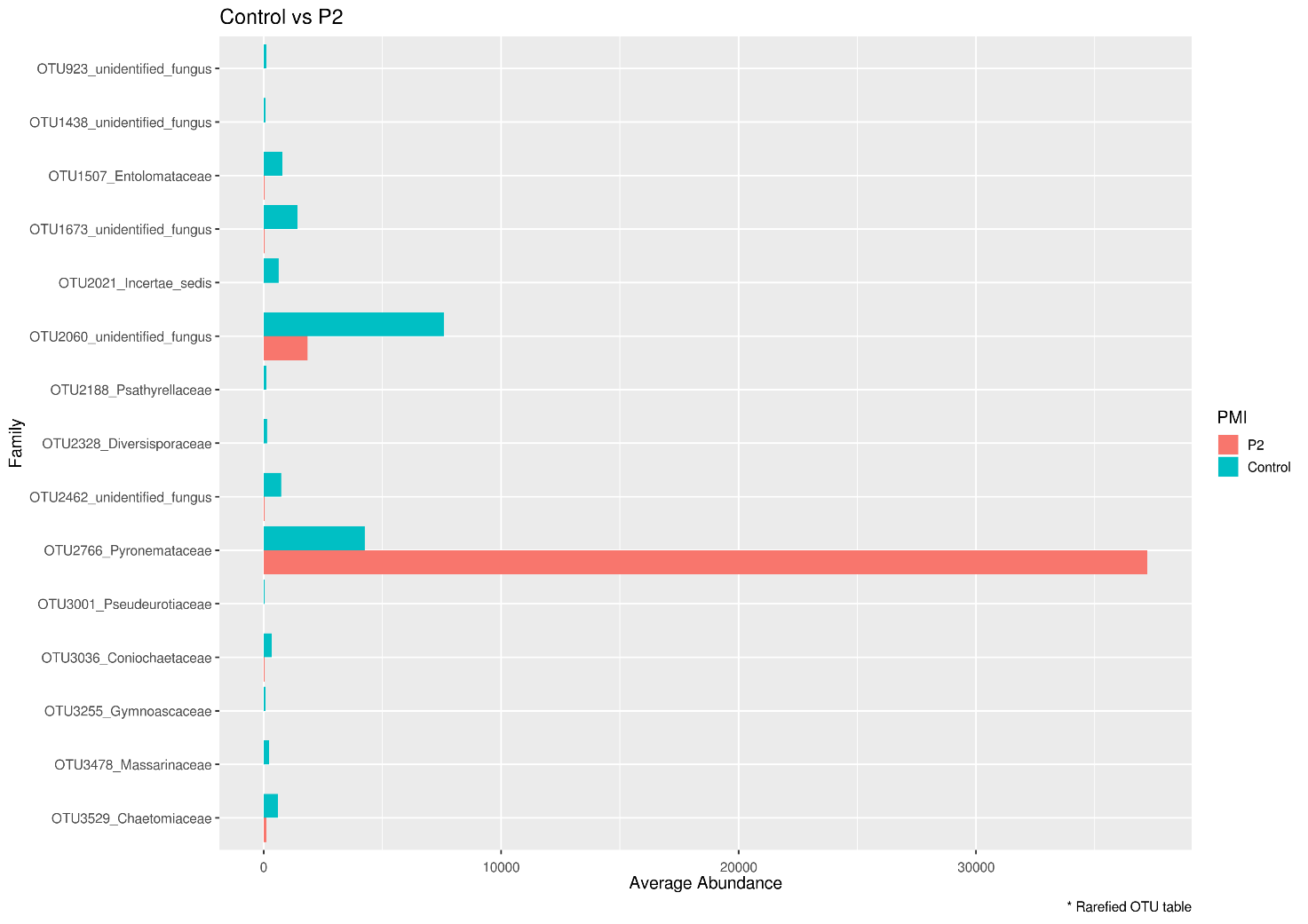


**Supplementary Figure S9.** Shifts in the average abundances of the basal communities that were statistically significant between the control (C1) and the grave soil collected after two-months PMI (P2) in Library B.


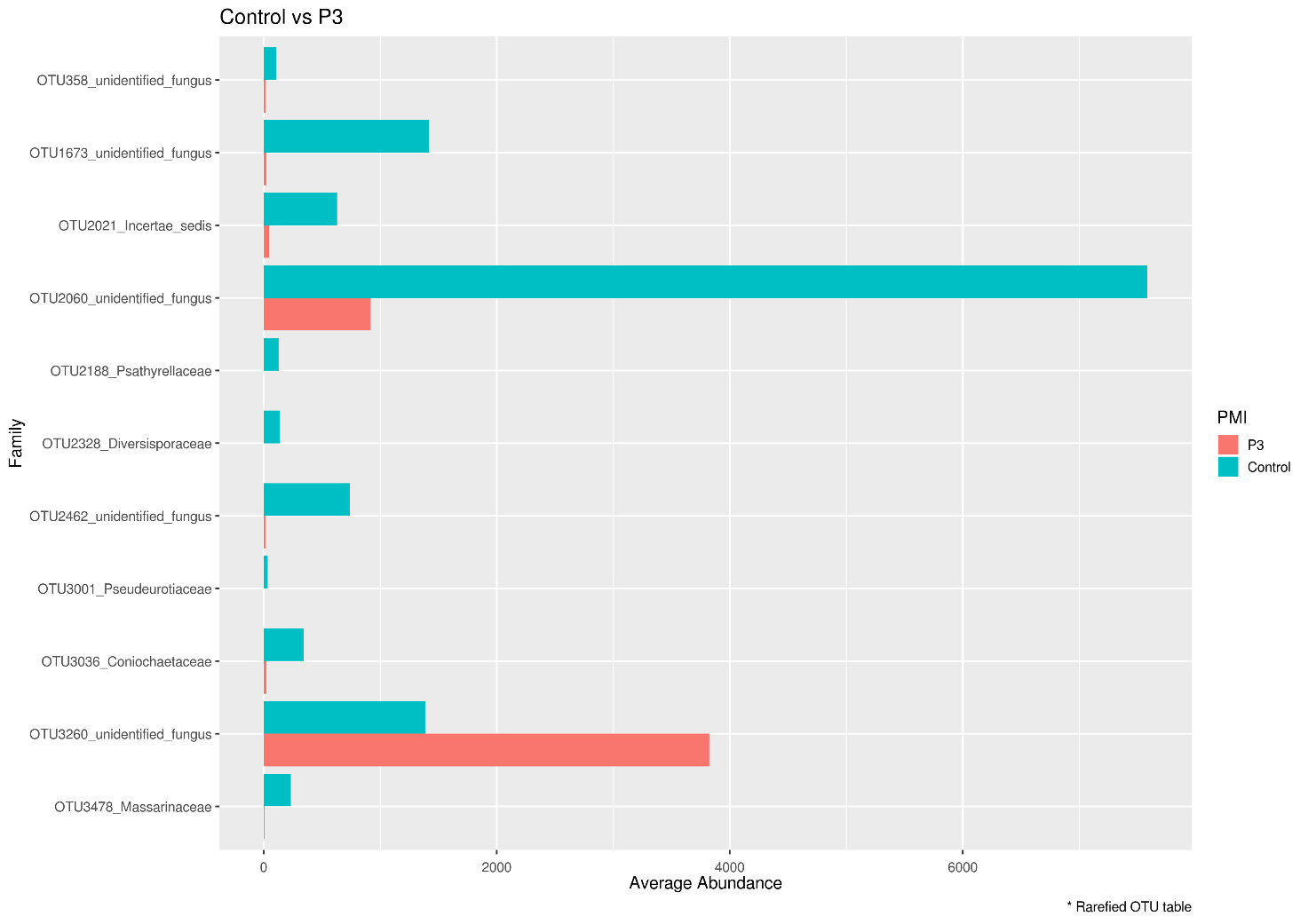


**Supplementary Figure S10.** Shifts in the average abundances of the basal communities that were statistically significant between the control (C1) and the grave soil collected after four-months PMI (P3) in Library B.


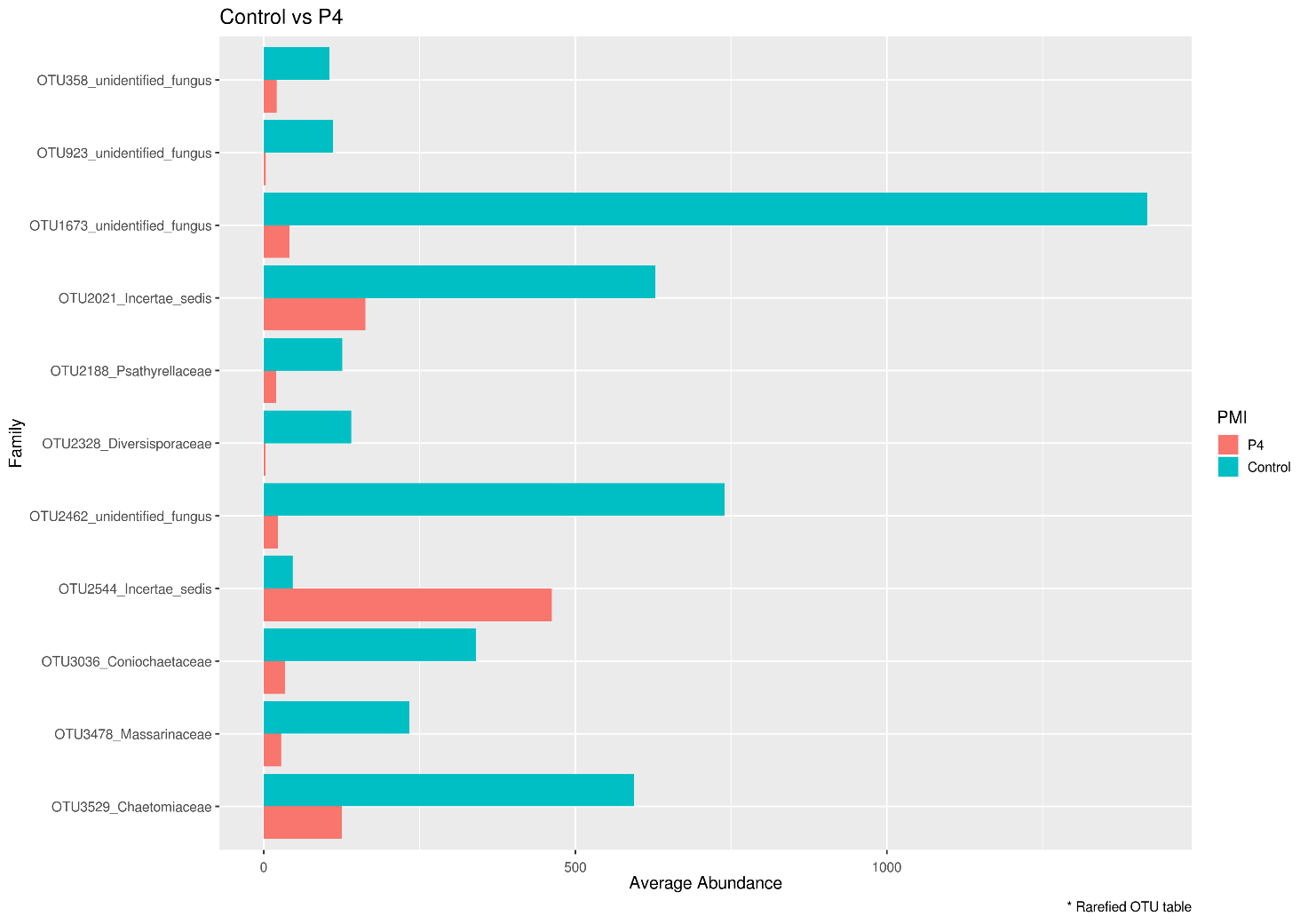


**Supplementary Figure S11**. Shifts in the average abundances of the basal communities that were statistically significant between the control (C1) and the grave soil collected after six-months PMI (P4) in Library B.
